# Minocycline reduces inflammatory response and cell death in a S100B retina degeneration model

**DOI:** 10.1186/s12974-020-02012-y

**Published:** 2020-12-14

**Authors:** Pia Grotegut, Natarajan Perumal, Sandra Kuehn, Andreas Smit, H. Burkhard Dick, Franz H. Grus, Stephanie C. Joachim

**Affiliations:** 1 Experimental Eye Research Institute, University Eye Hospital, Ruhr-University Bochum, In der Schornau 23-25, 44892 Bochum, Germany; 2 Experimental and Translational Ophthalmology, Department of Ophthalmology, University Medical Center of the Johannes Gutenberg University Mainz, Mainz, Germany

**Keywords:** S100B, Microglia, Minocycline, Retina, Retinal ganglion cells, Mitochondrial dysfunction, Glycolysis

## Abstract

**Background:**

Previous studies noted that intravitreal injection of S100B triggered a glaucoma-like degeneration of retina and optic nerve as well as microglia activation after 14 days. The precise role of microglia in our intravitreal S100B model is still unclear. Hence, microglia were inhibited through minocycline. The aim is to investigate whether microglia have a significant influence on the degeneration process or whether they are only a side effect in the model studied here.

**Methods:**

Minocycline was applied daily in rats by intraperitoneal injection using two different concentrations (13.5 mg/kg body weight, 25 mg/kg body weight). One day after treatment start, S100B or PBS was intravitreally injected in one eye per rat. The naïve groups received no injections. This resulted in a total of five groups (naïve *n* = 14, PBS *n* = 14, S100B *n* = 13, 13.5 mg/kg mino *n* = 15, 25 mg/kg mino *n* = 15). At day 14, electroretinogram measurements were performed, followed by immunofluorescence and label-free quantitative proteomics analysis. The focus of these investigations was on the survival of RGCs as well as their axons, the response of the microglia, and the identification of further pathological modes of action of S100B.

**Results:**

The best signal transmission was detected via ERG in the 13.5 mg/kg mino group. The inhibition of the microglia protected optic nerve neurofilaments and decreased the negative impact of S100B on RGCs. However, the minocycline treatment could not trigger complete protection of RGCs. Furthermore, in retina and optic nerve, the minocycline treatment reduced the number and activity of S100B-triggered microglia in a concentration-dependent manner. Proteomics analysis showed that S100B application led to numerous metabolic functions and cellular stress, mainly an increased inflammatory response, glycolysis, and mitochondrial dysfunction, which caused oxidative stress in the retina. Importantly, the protective capability of lower dose of minocycline was unraveled by suppressing the apoptotic, inflammatory, and the altered metabolic processes caused by S100B insult in the retina.

**Conclusion:**

Intravitreally injected S100B not only led to a pro-inflammatory microglial reaction, but also a mitochondrial and metabolic dysfunction. Also, these results suggest that an excessive microglial response may be a significant degenerative factor, but not the only trigger for increased cell death.

**Supplementary Information:**

The online version contains supplementary material available at 10.1186/s12974-020-02012-y.

## Background

S100B is a glial stress protein expressed by macroglia, especially astrocytes [1]. In patients with neuronal diseases, such as Alzheimer’s disease or multiple sclerosis, it is known that S100B levels in the cerebrospinal fluid are increased [2, 3]. Also, patients with glaucoma demonstrate an increase of autoantibodies against S100B in their tear fluid [4]. Hence, the role of S100B was investigated in glaucoma models [5, 6]. Systemic application of this protein led to retinal ganglion cell (RGC) and optic nerve damage in rats. In a recent study, intravitreal injection of S100B in rats induced a fast optic nerve degeneration, already after 3 days. Fourteen days after intravitreal injection, RGCs were also damaged, which indicates that the intravitreal S100B model is a glaucoma-like model [7]. In conjunction with these results, possible pathological mechanisms were investigated. An increased inflammatory response was detected 14 days after S100B injection. This inflammatory response included an increased number of retinal transcription factor nucleus factor-kappa B (NFκB) signals and an upregulated pro-inflammatory microglia response in retina and optic nerve [8]. It is known that S100B induces neuronal damage by activating the NFκB-dependent signal pathway [9], leading to a microglia activation in a pro-inflammatory manner. Therefore, we hypothesize that an uncontrolled microglial response is responsible for the strong neuronal damage in this glaucoma-like model. Hence, microglia and other immunological processes are described as possible degenerative players in this intravitreal S100B model and others, but it is unknown if they take part in degeneration in these models or if they are just an epiphenomenon [10, 11]. Therefore, the inhibition of microglia response might help to answer this question.

One possibility to inhibit the microglia is to use the tetracycline derivative antibiotic minocycline. Minocycline can increase the RNA expression of anti-apoptotic proteins in human trabecular meshwork cells and the astrocytes in the optic nerve head [12]. Also, it prevents the upregulation of caspases 1 and 3 and inhibits the release of cytochrome c in animal models [13, 14]. A very important characteristic is the anti-inflammatory quality of minocycline. These are not related to its microbial properties, but rather due to its inhibition of microglia cells [15]. The inhibition of microglia and its protecting effect on retinal cells was already noted in several glaucoma models [16–18].

To get a detailed analysis of the induced mechanisms, immunofluorescence and intensive proteome analyses were performed to identify further degenerative processes caused by S100B. Proteomics has proven to be an essential platform in ophthalmic research, especially in retinal-associated diseases owing to the advancement in the mass spectrometry technology that facilitated identification of potential disease markers for subsequent diagnosis and prognosis, as demonstrated by many previous studies, including several of ours [19–22]. Therefore, label-free quantitative proteomics strategy was employed to comprehensively characterize the retinal proteome in an experimental model of S100B-induced retinal insult as well as to evaluate the efficacy of minocycline treatment paradigm.

In this study, we examined the effect of minocycline in the intravitreal S100B model. We could demonstrate that minocycline treatment inhibited the microglia response and decreased the apoptosis rate in S100B-treated retinas as well as in optic nerves. Also, approximately 20% of the RGCs damaged by S100B and most of the damaged optic nerve neurofilaments were protected trough minocycline. Also, this study revealed alterations in the major metabolic functions, cellular stress, and corresponding dysfunctions, namely, mitochondrial dysfunction, apoptosis, inflammation, and neurodegenerative processes due to S100B-mediated insult and protective characteristics of minocycline in retina.

With this study, we could accurately identify the pathological properties of a high extracellular S100B concentration and determine the protective functions of minocycline in different concentrations.

## Material and methods

### Animals

All animal experiments were carried out in accordance with the ARVO Statement for Use of Animals in Research and the animal care committee of North Rhine-Westphalia (Germany; approval number 84-02.04.2013.A442). Male Wistar rats from Charles River (376–400 g; Sulzfeld, Germany) were used in this study, they had access to chow and water ad libitum on a 12 h:12 h light-dark cycle. At regular intervals, investigations of possible neurological deficits and eye damage were carried out and animals were weighed daily.

### Intraocular S100B injection

**Fig. 1:**
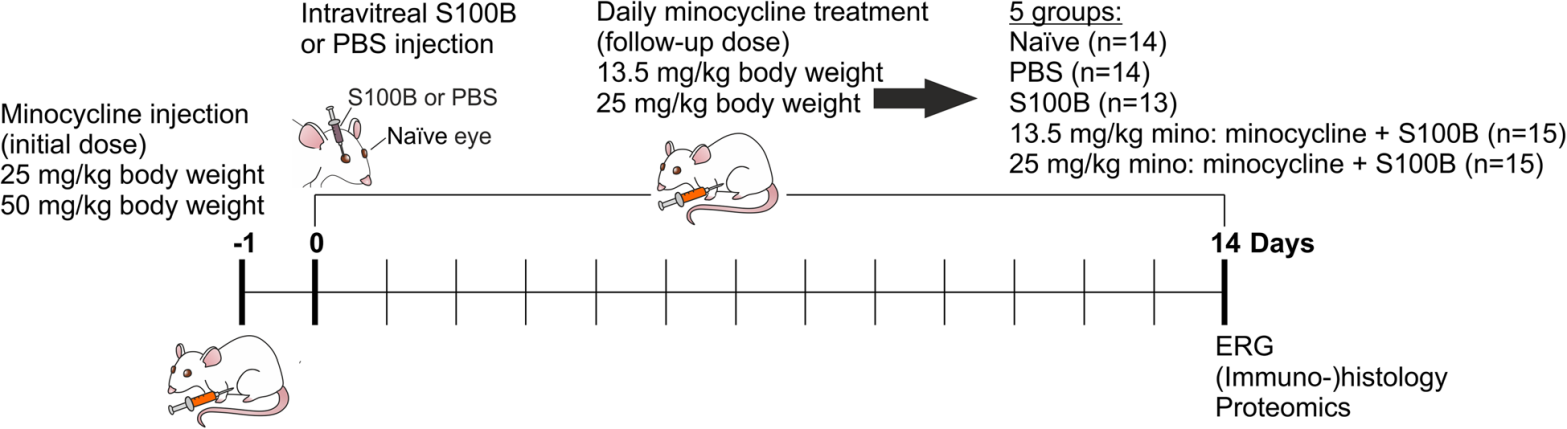
Study timeline. In this study, two different concentrations of minocycline (25 mg/kg body weight; 50 mg/kg body weight) were intraperitoneally injected at day -1. S100B or its solvent PBS were intravitreally applied at day 0. The untreated eyes were used as naïve controls. Minocycline was then administered daily (13.5 mg/kg body weight; 25 mg/kg body weight) from day 1 to 14. Five groups were generated in this study: naïve: untreated (*n* = 14); PBS: intravitreal PBS injection (*n* = 14); S100B: intravitreal S100B injection (*n* = 13); 13.5 mg/kg mino: low minocycline dose of 13.5 mg/kg + S100B (*n* = 15); 25 mg/kg mino: high minocycline dose of 25 mg/kg + S100B (*n* = 15). After 14 days, Electroretinogramm (ERG) measurements were carried out and the eyes and optic nerves were obtained for further analysis, including (immuno-)histology and proteomics

The intraocular S100B injections were carried out as described previously [7, 8]. 0.4 μg S100B solution was injected (Sigma Aldrich, St. Louis, MO, USA) in one eye (43 eyes in total). The control group received 2 μl of phosphate-buffered saline (PBS; Biochrome, Berlin, Germany, *n* = 14 eyes). The contralateral eyes were used as naïve controls (*n* = 14 eyes; Fig. 1a).

### Minocycline treatment

Thirty rats from the S100B group were treated with minocycline (Sigma Aldrich). This treatment was started 1 day before the intraocular injection occurred. These 30 rats were separated in two groups, the 13.5 mg/kg minocycline (13.5 mg/kg mino, *n* = 15) and 25 mg/kg minocycline groups (25 mg/kg mino, *n* = 15). Furthermore, the minocycline treatment was separated into an initial starting dose and follow-up doses. The initial dose was only given once on day -1 (1 day before S100B injection, Fig. 1). The 13.5 mg/kg mino group received 25 mg/kg body weight as an initial dose. As a follow-up dose, these animals received 13.5 mg/kg body weight intraperitoneally daily. The 25 mg/kg mino group received an initial dose of 50 mg/kg body weight and follow-up doses of 25 mg/kg body weight.

### Electroretinogram recordings

Electroretinogram (ERG) measurements were performed as previously described [7, 23]. A full-field flash electroretinography (HMsERG system; OcuScience, Henderson, NV, USA) was used to analyze the retinal function [24]. Recording electrodes were placed on the dilated and anesthetized eyes. Scotopic flash series were delivered to the eyes with flash intensities at 0.1, 0.3, 1, 3, 10, and 25 cd/m^2^ and readings recorded. To evaluate the data, the ERGView 4.380R software (OcuScience) was used. A low-pass filter (150 Hz) was necessary for the evaluation. At 0.1 cd/m^2^, the photopic negative response (PhNR) was also evaluated by measuring the lowest point of the PhNR to the baseline. The amplitude of the a- and b-wave, as well as the PhNR, was exported to Excel (Microsoft Corp., Redmond, WA, USA) for further analysis.

### Preparation of retina and optic nerve sections

After 14 days, eyes (*n* = 6/group) and optic nerves (*n* = 6/group) were obtained and prepared for histological cross-sections and longitudinal sections. The eyes were fixed for 1 h in 4% paraformaldehyde solution (Merck, Burlington, MA, USA), while optic nerves were fixed for two hours. Then, the tissue was cryo-conserved in 30% sucrose overnight and frozen embedded in NEG-50 Tissue-Tek medium (Thermo Fisher Scientific, Cheshire, UK). Optic nerve longitudinal sections (4 μm) and retinal cross-sections (10 μm) were cut using a cryostat (Thermo Fisher Waltham, MA, USA).

### Immunofluorescence staining of retina and optic nerve sections

Immunofluorescence stainings were carried out as previously described [5, 7]. Retina (6 sections/animal, *n* = 6/group) and optic nerve (6 sections/animal, *n* = 6/group) sections were blocked with a mixture of 10–20% serum, 0.1–0.2% Triton X-100 (Sigma-Aldrich), and PBS (Biochrome, Schaffhausen, Switzerland). The used primary antibodies (Table 1) were diluted in the same mixture and incubated at room temperature overnight. The sections were then incubated with Cy3/AlexaFluor 555- or AlexaFluor 488-labeled secondary antibodies in the same mixture (Table 1). The nuclei were stained with DAPI (0.01 μg) for 5 min. In the last step, the sections were covered with the Shandon mount (Thermo Fisher Scientific). For the evaluation, four images per retina (2 peripheral and 2 central) and three images per optic nerve (proximal, middle, and distal) were taken with an Axio Imager M2 microscope (Zeiss, Oberkochen, Germany). This enabled almost the entire retinal cross-section to be used for the evaluation. In regard to RBPMS^+^ RGCs, the complete cross-section was evaluated using an Axio Imager M2 microscope.

**Table 1:** Primary and secondary antibodies used for immunofluorescence

Primary antibodies			Secondary antibodies		
Antibody	Company	Dilution	Antibody	Company	Dilution
**Retinal cross-sections**					
Brn-3a	Santa Cruz	1:100	Donkey anti-goat Alexa Fluor 488	Abcam	1:400
Cleaved caspase 3	Sigma-Aldrich	1:300	Donkey anti-rabbit Alexa Fluor 555	Invitrogen	1:400
ED1	Millipore	1:250	Goat anti-mouse Alexa Fluor 488	Invitrogen	1:600
GFAP	Millipore	1:400	Donkey anti-chicken Cy3	Millipore	1:500
Iba1	Wako	1:500	Goat anti-rabbit IgG Cy3	Linaris	1:300
PKM	Abcam	1:500	Donkey anti-rabbit Alexa Fluor 555	Invitrogen	1:400
RBPMS	Millipore	1:300	Goat anti-rabbit Alexa Fluor 488	Millipore	1:500
S100B	Novus Biologicals	1:100	Goat anti-rabbit Alexa Fluor 488	Millipore	1:500
Tmem119	Synaptic System	1:500	Goat anti-mouse Alexa Fluor 488	Invitrogen	1:600
**Longitudinal optic nerve sections**					
Cleaved caspase 3	Sigma-Aldrich	1:100	Donkey anti-rabbit Alexa Fluor 555	Invitrogen	1:400
ED1	Millipore	1:200	Goat anti-mouse Alexa Fluor 555	Invitrogen	1:500
GFAP	Millipore	1:400	Donkey anti-chicken Cy3	Millipore	1:500
Iba1	Wako	1:400	Goat anti-rabbit Alexa Fluor 488	Invitrogen	1:500
S100B	Novus Biologicals	1:100	Donkey anti-rabbit Alexa Fluor 555	Invitrogen	1:400
SMI-32	Biolegend	1:2000	Goat anti-mouse Alexa Fluor 555	Invitrogen	1:500
Tmem119	Synaptic System	1:500	Goat anti-mouse Alexa Fluor 488	Invitrogen	1:600

The identified Iba1^+^-cell bodies were counted in all images. In regard to Tmem119 and ED1, the co-localization with Iba1 was evaluated. The signals of Iba1 and ED1 were counted in the ganglion cell layer (GCL), inner plexiform layer (IPL), and inner nuclear layer (INL). Also, cleaved caspase3^+^ cells were only counted when they were co-localized with Brn-3a signals in the GCL. In the optic nerve, cleaved caspase3^+^ cells were counted when they were co-localized with DAPI signals.

GFAP, S100B, and pyruvate kinase (PKM) signals in retina and optic nerve were investigated via area analysis using ImageJ [25]. It should be noted that the antibody for S100B does not mark the injected S100B. We assume that the injected S100B degrades after a few days. The displayed S100B analysis examines the S100B expressed by astrocytes. Therefore, it was used as a marker for the astrocytes [26]. All images were converted into gray scale (32-bit). Concerning GFAP, after background subtraction (55.32), the lower threshold was set at 8.62 and upper threshold at 36.95 for all retinal sections. Regarding the optic nerve sections, background subtraction was 40.7, while the lower threshold was 6.01 and the upper one 49.36. For the S100B staining in the retina, the background subtraction was 58.35, while the lower threshold was 7.56 and the upper one 33.84. The background subtraction for the optic nerve sections was 48.25, while the lower threshold was 2.58 and the upper one 20.74. For PKM analysis, the background subtraction was 55.5. The low threshold was set to 10.1, while the upper threshold was 84.1.

SMI-32-labeled neurofilaments in optic nerves were scored from 0 = intact up to 2 = destroyed in a masked fashion, in 0.5 intervals using an established scoring system [25].

### Optic nerve histology

The structures of the optic nerve were also evaluated after hematoxylin and eosin (H&E; both Merck) staining. Three pictures per HE-stained optic nerve (proximal, middle, and distal, *n* = 6/group) were taken at × 400 magnification. All pictures from the optic nerve were scored from 0 = no infiltration up to 4 = massive infiltration of the optic nerve parenchyma and nodule infiltration using 1 intervals [27].

### Statistical analysis of histological evaluations

Statistical analysis was performed using Statistica (Version 13, Dell Technologies, Round Rock, TX, USA). Regarding ERG recordings and histological data, groups were compared by ANOVA followed by Tukey’s post hoc test. All results are presented as mean ± standard error mean (SEM) with *p < 0.05, **p < 0.01, and ***p < 0.001 compared to the naïve group, ^#^*p* < 0.05, ^##^*p* < 0.01, and ^###^*p* < 0.001 compared to the PBS group, and ^¥^*p* < 0.05, ^¥¥^*p* < 0.01, and ^¥¥¥^*p* < 0.001 compared to the S100B group.

### Sample preparation for proteomic analysis

Retinal samples of the following groups were prepared for proteomic analysis: PBS, S100B, 13.5 mg/kg mino, 25 mg/kg mino (*n* = 6/group; supplemental table 1). Two retinas per group were pooled to get one biological replicate. A total of three biological replicates were used in this study [28]. Rat retinas were carefully isolated from the eye, rinsed in ice-cold PBS, and frozen in tubes at − 80 °C. Briefly, two isolated retinal tissues were thawed, pooled, weighed, and mechanical pre-homogenized with a scalpel and a metal homogenizer (Neolab, Heidelberg, Germany), followed by a chemical protein extraction with 1 ml 0.1% SDS added to 0.05 g retina samples. Next, the samples were homogenized in 0.1% SDS reagent with an ultrasonicator on ice for approximately 1 min for 3 cycles with a 1-min interval between the cycles. The homogenate was centrifuged at 10,000*g* for 5 min (4 °C). The supernatant was subjected to sample cleaning and concentrated using Amicon Ultra 0.5-mL centrifugal filters with 3K cutoff (Merck Millipore, Carrigtwohill, Ireland). The protein concentration of the obtained eluate was determined using BCA Protein Assay Kit (Pierce, Rockford, IL).

The designated samples were then subjected to one-dimensional gel electrophoresis (1DE) employing precast NuPAGE 4-12% Bis-Tris 10-well mini protein gels (Invitrogen, Karlsruhe, Germany) with 2-[N-morpholino] ethanesulfonic acid (MOPS) running buffer under reducing conditions at a constant voltage of 150 V at. Pre-stained protein standard, SeeBlue Plus2 (Invitrogen, Karlsruhe, Germany), was used as a molecular mass marker, and gels were fixed and stained with Colloidal Blue Staining Kit (Invitrogen, Karlsruhe, Germany), as per manufacturer’s instructions. Protein bands of two wells/sample were combined to get three replicates/sample. They were excised (25 bands per replicate) and were destained in 100 mM ammonium bicarbonate/acetonitrile (1:1, vol/vol) buffer. The protein/gel pieces were reduced in fresh prepared 10 mM dithiothreitol in 50 mM ammonium bicarbonate buffer, and alkylated in 55 mM iodoacetamide in 50 mM ammonium bicarbonate buffer prior to in-gel trypsin digestion employing sequence grade-modified trypsin (Promega, Madison, USA), as described in detail by Perumal et al. [29]. Peptides extracted from trypsin digestion were purified with SOLAμ SPE HRP plates (Thermo Fisher Scientific, Rockford, USA) according to the manufacturer’s instructions. The resulting peptide eluate was concentrated to dryness in a centrifugal vacuum evaporator and dissolved in 10 μl 0.1% trifluoroacetic acid (TFA) for LC-MS/MS analysis.

### Discovery proteomics strategy

Label-free quantitative proteomics analysis was performed on a liquid chromatography-electrospray ionization-linear ion trap-Orbitrap XL (LC-ESI-LTQ-Orbitrap) MS system (Thermo Scientific, Bremen, Germany), and the relevant details pertaining to the LC gradients and the settings are as described in detail elsewhere [22, 29].

The acquired continuum MS spectra were analyzed by MaxQuant computational proteomics platform version 1.6.3.3. It is a built-in Andromeda search engine for peptide and protein identification [30–34]. The tandem MS spectra were searched against *Homo sapiens*; Date: 17 Oct 2018; 20410 proteins) and *Rattus norvegicus* (Date: 17 Oct 2018; 8050 proteins) databases, using standard settings with peptide mass tolerance of ± 30 ppm and fragment mass tolerance of ± 0.5 Da, with ≥ 6 amino acid residues and only “unique plus razor peptides” that belong to a protein were chosen [30]. A target-decoy-based false discovery rate (FDR) for peptide and protein identification was set to 0.01. The summary of MaxQuant parameters employed in the current analyses is tabulated in supplemental table 2a.

### Bioinformatics and functional annotation and pathway analyses

The output of the generated “proteingroups.txt” data from the MaxQuant analysis was utilized for subsequent statistical analysis with Perseus software (version1.6.5.0). First, all protein intensities were log_2_-transformed, the data were filtered with minimum of three valid values in at least one group, and the missing values were imported by replacing from normal distribution (width: 0.3; down shift. 1.8) using the standard settings in Perseus [34]. For statistical evaluation, two-sided Student’s *t* test was utilized for the group comparison with *p* < 0.05 to identify the significantly differentially abundant proteins. Unsupervised hierarchical clustering analysis of the differentially abundant proteins was conducted based on the log_2_ fold change values according to Euclidean distance (linkage = average; preprocess with k-means) and elucidated in a heat map. Venn diagrams were generated utilizing the InteractiviVenn tool (http://www.interactivenn.net/). The list of the differentially abundant proteins was tabulated in Excel and their gene names were used for subsequent functional annotation and pathway analyses employing Ingenuity Pathway Analysis (v01-04, IPA; Ingenuity QIAGEN Redwood City, CA) (https://www.qiagenbioinformatics.com/products/ingenuity-pathway-analysis) [35]. IPA analyses elucidated the molecular types, gene ontology cellular component (GOCC) terms, protein-protein interaction (PPI) networks, canonical pathways, upstream regulators and top disease, and functions associated with the proteins identified to be differentially abundant. In PPI networks, protein molecules are represented by their corresponding gene names and only PPIs that were experimentally observed and had direct and indirect interactions were used.

## Results

### Slight improvement of retinal function after treatment

Retinal functionality was investigated via ERG recordings after 14 days. The a-wave amplitude of the minocycline animals demonstrated the highest values at all light intensities (supplemental fig. 1a). However, significant differences between the five groups were only observed at 0.1, 10, and 25 cd.s/m^2^ (Table 2). At 0.1 cd.s/m^2^, the 13.5 mg/kg mino group demonstrated much higher values than the PBS (*p* = 0.01) and the S100B group (*p* = 0.01). Also, the 25 mg/kg mino group demonstrated higher amplitudes than the PBS (*p* = 0.01) and the S100B group (*p* = 0.006). At 10 cd.s/m^2^, the 13.5 mg/kg mino group showed higher values than the S100B group (*p* = 0.04). At 25 cd.s/m^2^, the amplitudes of the S100B animals were significantly reduced compared to the naïve group (*p* = 0.04). The 13.5 mg/kg mino animals displayed higher amplitudes than the S100B animals (*p* = 0.02). All other groups showed no differences at all light intensities.

**Table 2:** Data of a- and b-wave amplitudes as well as PhNR recorded from naïve, PBS, S100B, 13.5 mg/kg mino, and 25 mg/kg mino animals. Values of light flash intensity (cd.s/m^2^) are displayed as mean ± SEM. *p* values < 0.05 are shown in bold

	Cd.s/m^2^	Mean ± SEM [μV]					*P*-values									
		Naïve	PBS	S100B	13.5 mg/kg mino	25 mg/kg mino	Naïve vs. PBS	Naïve vs. S100B	Naïve vs. 13.5 mg/kg mino	Naïve vs. 25 mg/kg mino	PBS vs. S100B	PBS vs. 13.5 mg/kg mino	PBS vs. 25 mg/kg mino	S100B vs. 13.5 mg/kg mino	S100B vs. 25 mg/kg mino	13.5 vs. 25 mg/kg mino
**A-wave**	0.1	58.5 ± 5.6	42.3 ± 7.1	40.3 ± 6.2	85.7 ± 11.6	73.2 ± 10.1	0.66	0.56	0.19	0.74	1.00	**0.01**	0.10	**0.001**	0.07	0.83
	0.3	80.6 ± 15.4	70.4 ± 8.1	68.8 ± 10.2	98.2 ± 9.8	81.7 ± 6.0	0.96	0.93	0.75	1.00	1.00	0.35	0.94	0.29	0.90	0.79
	1.0	112.1 ± 13.4	92.3 ± 6.5	74.5 ± 3.8	113.9 ± 16.2	95.5 ± 5.1	0.65	0.10	1.00	0.78	0.74	0.58	1.00	0.08	0.60	0.71
	3.0	120.3 ± 12.2	114.0 ± 5.6	85.0 ± 7.0	126.5 ± 17.5	102.7 ± 3.8	0.99	0.15	0.99	0.76	0.32	0.92	0.94	0.07	0.76	0.51
	10.0	150.3 ± 17.2	131.8 ± 6.2	101.7 ± 5.9	152.4 ± 15.4	136.7 ± 9.5	0.80	0.054	1.00	0.92	0.40	0.73	1.00	**0.04**	0.26	0.88
	25.0	157.1 ± 13.6	128.7 ± 8.0	105.7 ± 5.9	164.6 ± 20.4	134.7 ± 7.1	0.49	**0.04**	1.00	0.70	0.68	0.26	1.00	**0.02**	0.47	0.43
**B-wave**	0.1	223.8 ± 31.1	203.5 ± 15.4	179.7 ± 6.7	251.8 ± 22.4	246.1 ± 20.4	0.96	0.57	0.87	0.94	0.93	0.49	0.60	0.14	0.19	1.00
	0.3	272.2 ± 24.9	238.0 ± 20.6	208.0 ± 8.2	305.0 ± 19.6	281.0 ± 23.6	0.76	0.20	0.78	1.00	0.83	0.17	0.57	**0.02**	0.11	0.92
	1.0	325.1 ± 36.9	272.2 ± 14.0	208.5 ± 12.7	323.7 ± 33.1	306.7 ± 12.3	0.55	**0.02**	1.00	0.98	0.37	0.57	0.85	**0.02**	0.06	0.99
	3.0	322.8 ± 30.0	275.1 ± 16.1	244.4 ± 14.2	324.8 ± 36.4	291.6 ± 13.3	0.63	0.17	1.00	0.89	0.89	0.59	0.99	0.16	0.63	0.86
	10.0	357.0 ± 46.2	300.4 ± 8.7	376.4 ± 13.0	360.7 ± 52.6	373.6 ± 17.4	0.74	0.44	1.00	1.00	0.99	0.70	0.53	0.39	0.26	1.00
	25.0	377.5 ± 47.6	337.2 ± 14.5	277.0 ± 14.7	327.1 ± 42.6	324.6 ± 8.7	0.88	0.16	0.76	0.73	0.63	1.00	1.00	0.77	0.80	1.00
**PhNR**	0.1	− 24.2 ± 3.0	− 22.2 ± 1.7	− 11.7 ± 2.0	− 18.1 ± 3.3	− 20.8 ± 4.5	0.99	0.06	0.62	0.93	0.15	0.87	1.00	0.60	0.25	0.97

The analysis of the b-wave demonstrated significant differences at 0.3 and 1 cd.s/m^2^ (supplemental fig. 1b; Table 2). At 0.3 cd.s/m^2^, the 13.5 mg/kg mino group displayed significantly higher values than the S100B group (*p* = 0.02). The other groups demonstrated no differences. At 1 cd.s/m^2^, the b-wave amplitude of the S100B group was reduced in comparison to the naïve group (*p* = 0.02). A significant increase of the b-wave amplitude of the 13.5 mg/kg mino group were measured compared to the S100B group (*p* = 0.02).

The photopic negative response (PhNR) is a negative potential after the b-wave [36, 37], which was measured at 0.1 cd/m^2^ (supplemental fig. 1c; Table 2). No differences were observed between all groups.

In summary, minocycline appears to have a positive effect on electrical signal transmission.

### Mild protection of retinal ganglion cells

**Fig. 2:**
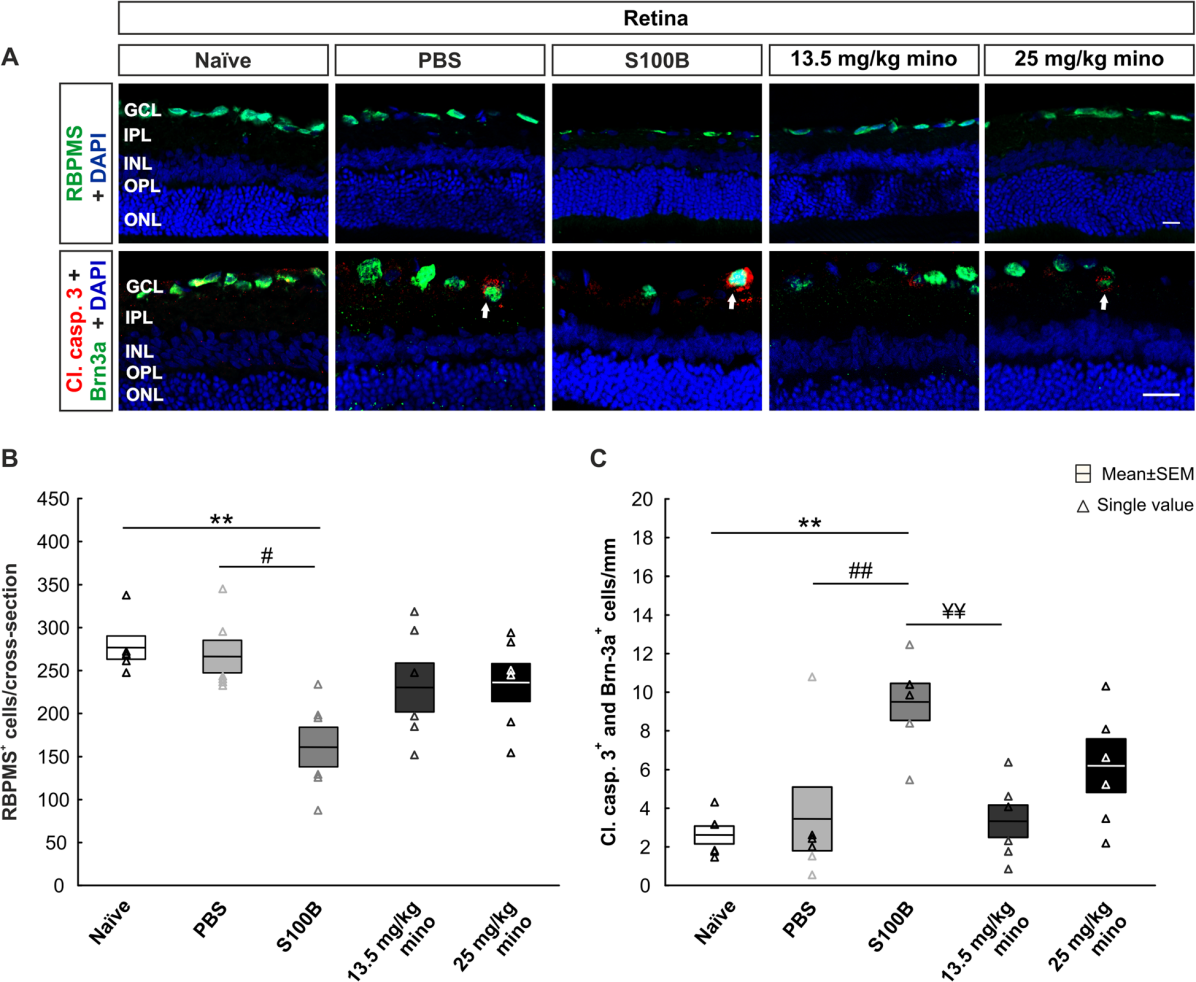
Protection of retinal ganglion cells. **a** RGCs were stained with the antibody RBPMS (green). Apoptotic (red, cl. caspase 3, arrows) RGCs (green, Brn3a) and cell nuclei (blue) were also labeled with specific antibodies on retinal cross-sections. **b** Intravitreally injected S100B triggered an RGC loss compared to the PBS and naïve group. Some RBPMS^+^ RGCs were protected via minocycline in comparison to the S100B group, but these differences were not significant. **c** A higher apoptotic rate of RGCs was noted in the S100B group compared to both controls. In contrast, minocycline treatment reduced the apoptotic rate in a dose-dependent manner. Abbreviations: GCL: ganglion cell layer, IPL: inner plexiform layer, INL: inner nuclear layer , OPL: outer plexiform layer, ONL: outer nuclear layer. Scale bar: 20 μm, *n* = 6/group,**p < 0.01 *vs*. naïve group, ^#^*p* < 0.01, ^##^*p* < 0.05 *vs.* PBS group, ^¥¥^*p* < 0.01 *vs.* S100B group

To analyze a possible neurodegeneration, cells were labeled with the specific RGC marker RBPMS. In order to identify RGCs in the apoptotic state, the RGCs were labeled with the RGC marker Brn-3a together with the apoptotic marker cleaved caspase 3 (Fig. 2a). The intravitreal S100B injection led to a significant loss of RBPMS^+^ RGCs compared to the naïve (*p* = 0.008) and the PBS group (*p* = 0.02; Fig. 2b; Table 3). All other groups did not show any significant differences between each other.

**Table 3:** RBPMS, cleaved caspase 3, Iba1, Tmem119, and ED1 cell counts (cells/mm in the retina and mm^2^ in the optic nerve), PKM, GFAP, and S100B area evaluations (%), as well as SMI-32 and H&E scores for immunofluorescence stains in naïve, PBS, S100B, 13.5 mg/kg mino, and 25 mg/kg mino animals. Values are displayed as mean ± SEM. *p* values < 0.05 are shown in bold

	Cell type	Mean ± SEM					*P*-values									
		Naïve	PBS	S100B	13.5 mg/kg mino	25 mg/kg mino	Naïve vs. PBS	Naïve vs. S100B	Naïve vs. 13.5 mg/kg mino	Naïve vs. 25 mg/kg mino	PBS vs. S100B	PBS vs. 13.5 mg/kg mino	PBS vs. 25 mg/kg mino	S100B vs. 13.5 mg/kg mino	S100B vs. 25 mg/kg mino	13.5 vs. 25 mg/kg mino
**Retina**	RBPMS^+^	276.8 ± 13.6	266.3 ± 19.0	161.1 ± 22.9	230.3 ± 28.5	236.0 ± 22.0	1.00	**0.008**	0.56	0.68	**0.02**	0.77	0.86	0.20	0.14	1.00
	Cl. casp. 3^+^ + Brn3a^+^	2.6 ± 0.5	3.5 ± 1.7	9.5 ± 1.0	3.3 ± 0.8	6.2 ± 1.4	1.00	**0.002**	1.00	0.20	**0.008**	1.00	0.45	**0.006**	0.27	0.40
	Iba1^+^	10.1 ± 1.0	11.7 ± 0.5	19.0 ± 0.8	11.1 ± 0.8	11.5 ± 1.0	0.66	**< 0.001**	0.90	0.74	**< 0.001**	1.00	1.00	**< 0.001**	**< 0.001**	1.00
	Tmem119 + Iba1^+^	9.7 ± 0.9	11.3 ± 0.4	18.6 ± 0.7	10.9 ± 0.7	10.9 ± 0.9	0.57	**< 0.001**	0.77	0.78	**< 0.001**	1.00	1.00	**< 0.001**	**< 0.001**	1.00
	Tmem119^-^ + Iba1^+^	3.0 ± 1.5	3.0 ± 1.5	3.0 ± 1.5	1.5 ± 0.9	1.5 ± 0.9	1.00	1.00	0.92	0.92	1.00	0.92	0.92	0.92	0.92	1.00
	ED1^+^ + Iba1^+^	1.3 ± 0.4	2.4 ± 0.9	5.2 ± 1.2	2.5 ± 1.4	2.4 ± 1.3	0.48	**< 0.001**	0.40	0.48	**0.002**	1.00	1.00	**0.002**	**0.002**	1.00
	PKM^+^	14.8 ± 4.8	14.9 ± 5.3	7.5 ± 1.9	10.1 ± 3.9	9.8 ± 3.6	1.00	**0.03**	0.29	0.24	**0.03**	0.26	0.22	0.81	0.86	1.00
	GFAP^+^	3.1 ± 0.4	3.7 ± 0.4	3.1 ± 0.4	3.1 ± 0.3	4.3 ± 0.6	0.90	1.00	1.00	0.38	0.90	0.86	0.88	1.00	0.38	0.33
	S100B^+^	9.3 ± 1.8	8.0 ± 2.1	7.7 ± 0.9	8.5 ± 2.8	9.5 ± 1.8	0.78	0.66	0.96	1.00	1.00	1.00	0.66	0.96	0.52	0.90
**Optic nerve**	SMI-32	0.7 ± 0.1	0.8 ± 0.1	1.3 ± 0.0	0.9 ± 0.1	1.0 ± 0.1	0.61	**< 0.001**	0.20	**0.02**	**0.003**	0.93	0.34	**0.02**	0.19	0.79
	Cl. casp. 3^+^	42.6 ± 5.1	43.1 ± 4.6	84.9 ± 8.6	45.0 ± 6.3	59.0 ± 9.2	1.00	**0.004**	1.00	0.53	**0.003**	1.00	0.51	**0.004**	0.10	0.63
	Iba1^+^	78.5 ± 6.0	83.7 ± 4.4	130.0 ± 9.3	72.4 ± 11.8	90.4 ± 10.6	1.00	**0.003**	1.00	0.87	**0.009**	0.90	0.98	**0.001**	**0.03**	0.61
	Tmem119^+^ + Iba1^+^	77.0 ± 6.1	80.7 ± 3.8	124.1 ± 9.2	70.2 ± 11.7	87.4 ± 10.1	1.00	**0.006**	0.98	0.91	**0.01**	0.91	0.98	**0.002**	**0.04**	0.63
	Tmem119^-^ + Iba1^+^	1.5 ± 0.9	3.0 ± 1.5	6.3 ± 3.0	2.2 ± 1.0	3.0 ± 1.9	0.97	0.35	1.00	0.97	0.69	1.00	1.00	0.51	0.69	1.00
	ED1^+^ + Iba1^+^	65.4 ± 10.6	85.7 ± 11.8	163.3 ± 14.0	85.1 ± 7.0	84.5 ± 10.0	0.69	**< 0.001**	0.70	0.73	**< 0.001**	1.00	1.00	**< 0.001**	**< 0.001**	1.00
	H&E	0.7 ± 0.2	1.0 ± 0.2	1.9 ± 0.2	0.8 ± 0.2	1.1 ± 0.2	0.83	**0.003**	0.98	0.66	**0.04**	0.99	1.00	**0.01**	0.08	0.92
	GFAP^+^	13.2 ± 1.2	13.7 ± 2.2	21.8 ± 2.2	18.8 ± 2.1	19.5 ± 2.0	1.00	**0.04**	0.30	0.20	0.06	0.38	0.26	0.82	0.92	1.00
	S100B^+^	9.6 ± 1.4	10.4 ± 1.7	8.3 ± 2.4	9.4 ± 1.6	7.3 ± 0.7	1.00	0.98	1.00	0.85	0.89	0.99	0.68	0.99	0.99	0.89

Regarding the cleaved caspase 3^+^ and Brn-3a^+^ cells, the S100B group showed significantly more apoptotic RGCs than the naïve (*p* = 0.002), the PBS (*p* = 0.008), and the 13.5 mg/kg mino group (*p* = 0.006; Fig. 2c; Table 3). Apart from this, the groups did not show any other significant differences between each other.

In summary, the minocycline treatment seems to reduce the number of apoptotic cells, saving some RGCs.

### Fewer apoptotic signals led to less neurofilament degeneration after minocycline treatment

**Fig. 3:**
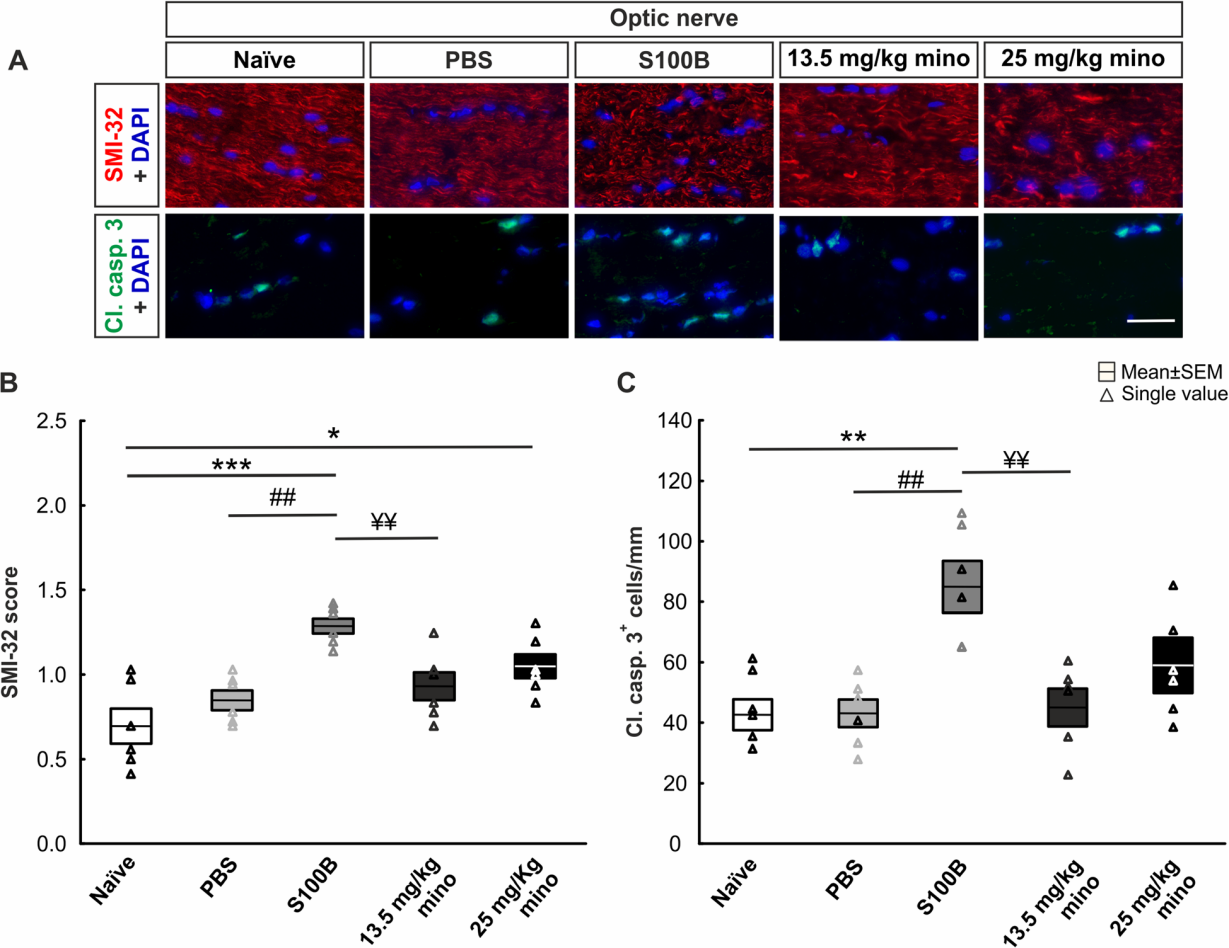
Protection of neurofilament through minocycline. **a** Optic nerve neurofilament was stained with SMI-32 (red), apoptotic cells with cleaved caspase 3 (green), and cell nuclei with DAPI (blue). **b** An increased SMI-32 score, indicating loss of optic nerve structure, was noted in the S100B group compared to both control groups. Minocycline application showed a dose-dependent prevention of optic nerve destruction. **c** A higher apoptotic rate was noted in the S100B group compared to both controls. Minocycline treatment reduced the number of cleaved caspase 3^+^ cells in a dose-dependent manner. Scale bar: 20 μm, *n* = 6/group, *p < 0.05, **p < 0.01, ***p < 0.001 *vs.* naïve group, ^##^*p* < 0.01 *vs.* PBS group, ^¥¥^*p* < 0.01 *vs.* S100B group

In order to identify degenerative processes in the optic nerve, the axonal neurofilament (SMI-32) and the apoptotic cells (cleaved caspase 3) were marked with immunofluorescence (Fig. 3a).

Regarding the SMI-32 evaluation, the S100B group showed the highest score (Fig. 3b; Table 3). Hence, neurofilaments were damaged. In comparison to the naïve (*p* < 0.001), PBS (*p* = 0.003), and 13.5 mg/kg mino group (*p* = 0.02), this value was significantly increased in the S100B group. All other groups did not show any significant differences between each other.

As observed in the retina, the injection of S100B led to a significant increase in cleaved caspase 3^+^ cells compared to naïve (*p* = 0.004), PBS (*p* = 0.003), and 13.5 mg/kg mino optic nerves (*p* = 0.004). In contrast, no differences were noted between the other groups (Fig. 3c; Table 3).

Also, in the optic nerve, the minocycline treatment seems to reduce the number of apoptotic cells, which might then lead to a preservation of the neurofilament structure.

### Reduced microglia activation in retina through minocycline treatment

**Fig. 4:**
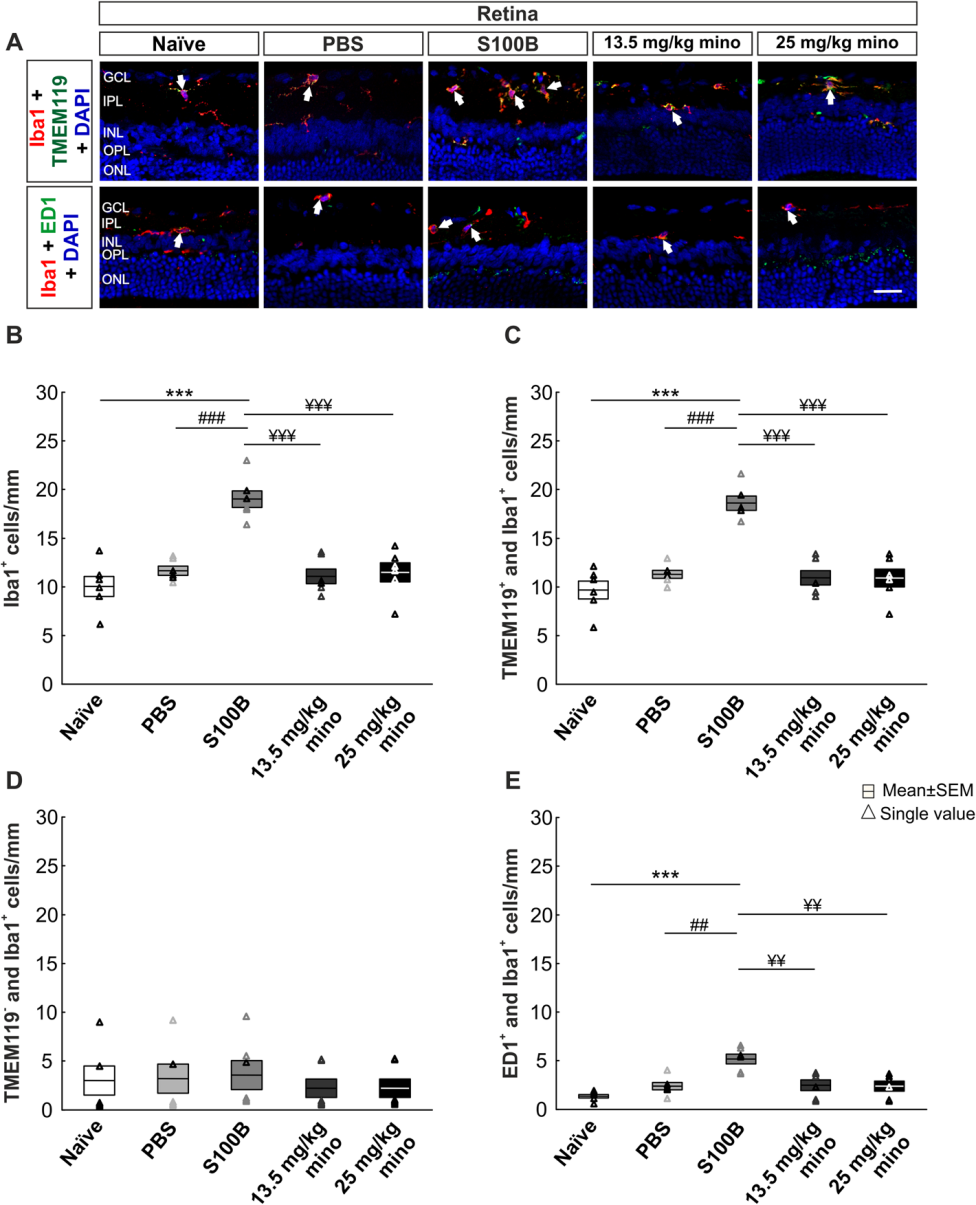
Diminished microglia activity in the retina. **a** Iba1 antibody (red) was used to label all phagocytes and combined with Tmem119 antibody (green) to distinguish between microglia (Tmem119^+^ and Iba1^+^) and recruited phagocytes (Tmem119^−^ and Iba1^+^). Active microglia/macrophages were identified by the co-localization of ED1 (green; arrows) and Iba1. **b** The number of Iba1^+^ cells was increased in the S100B group compared to both controls. Minocycline treatment reduced the S100B effect significantly. **c** The number of microglia (Tmem119^+^ and Iba1^+^) was upregulated in the S100B group compared to the naïve and PBS group. Both minocycline treatments significantly reduced this upregulation. **d** The number of phagocytes (Tmem119^−^ and Iba1^+^) was similar in all groups. **e** Also, higher active microglia/macrophage counts were noted in the S100B group in contrast to both control groups. ED1^+^ and Iba1^+^ cell numbers in the minocycline groups were reduced. Abbreviations: GCL: ganglion cell layer, IPL: inner plexiform layer, INL: inner nuclear layer, OPL: outer plexiform layer, ONL: outer nuclear layer. Scale bar: 20 μm, *n* = 6/group, ***p < 0.001 *vs.* naïve group, ^##^*p* < 0.01, ^###^*p* < 0.001 *vs.* PBS group, ^¥¥^*p* < 0.01, ^¥¥¥^*p* < 0.001 *vs.* S100B group

The effect of S100B as well as minocycline treatment on microglia was analyzed in the retina after 14 days (Fig. 4a). All phagocytic cells were identified with an Iba1 antibody. The co-localization with Tmem119 antibody was used to differentiate between resident microglia and recruited phagocytes [38].

In the S100B group, the number of phagocytic cells (Iba1^+^) was increased compared to the naïve (*p* < 0.001) and the PBS group (*p* < 0.001; Fig. 4b, Table 3). Furthermore, both minocycline groups demonstrated significantly lower phagocyte numbers compared to the S100B group (13.5 mg/kg mino: *p* < 0.001; 25 mg/kg mino: *p* < 0.001).

Regarding the number of microglia (Tmem119^+^ and Iba1^+^), a similar result was observed (Fig. 4c; Table 3). The number of microglia in the S100B group was increased compared to the naïve (*p* < 0.001), the PBS (*p* < 0.001), the 13.5 mg/kg mino (*p* < 0.001), and the 25 mg/kg mino group (*p* < 0.001).

In contrast, the number of infiltrated macrophages (Tmem119^−^ and Iba1^+^) was comparable in all groups (Fig. 4d; Table 3).

Regarding the number of cells with macrophage function (ED1^+^ and Iba1^+^), similar results were seen as in microglia counts. The number of active phagocytes was highly increased in the S100B group compared to the naïve (*p* < 0.001), the PBS (*p* = 0.002), and both minocycline groups (13.5 mg/kg mino: *p* = 0.002; 25 mg/kg mino: *p* = 0.002; Fig. 4e; Table 3). All other groups did not show any significant differences between each other.

Minocycline appears to suppress the S100B-induced activation of microglia.

### Minocycline reduced microglia response in the optic nerve

Antibodies against Iba1 and Tmem199 were also used in the optic nerve to distinguish between microglia and macrophages.

**Fig. 5:**
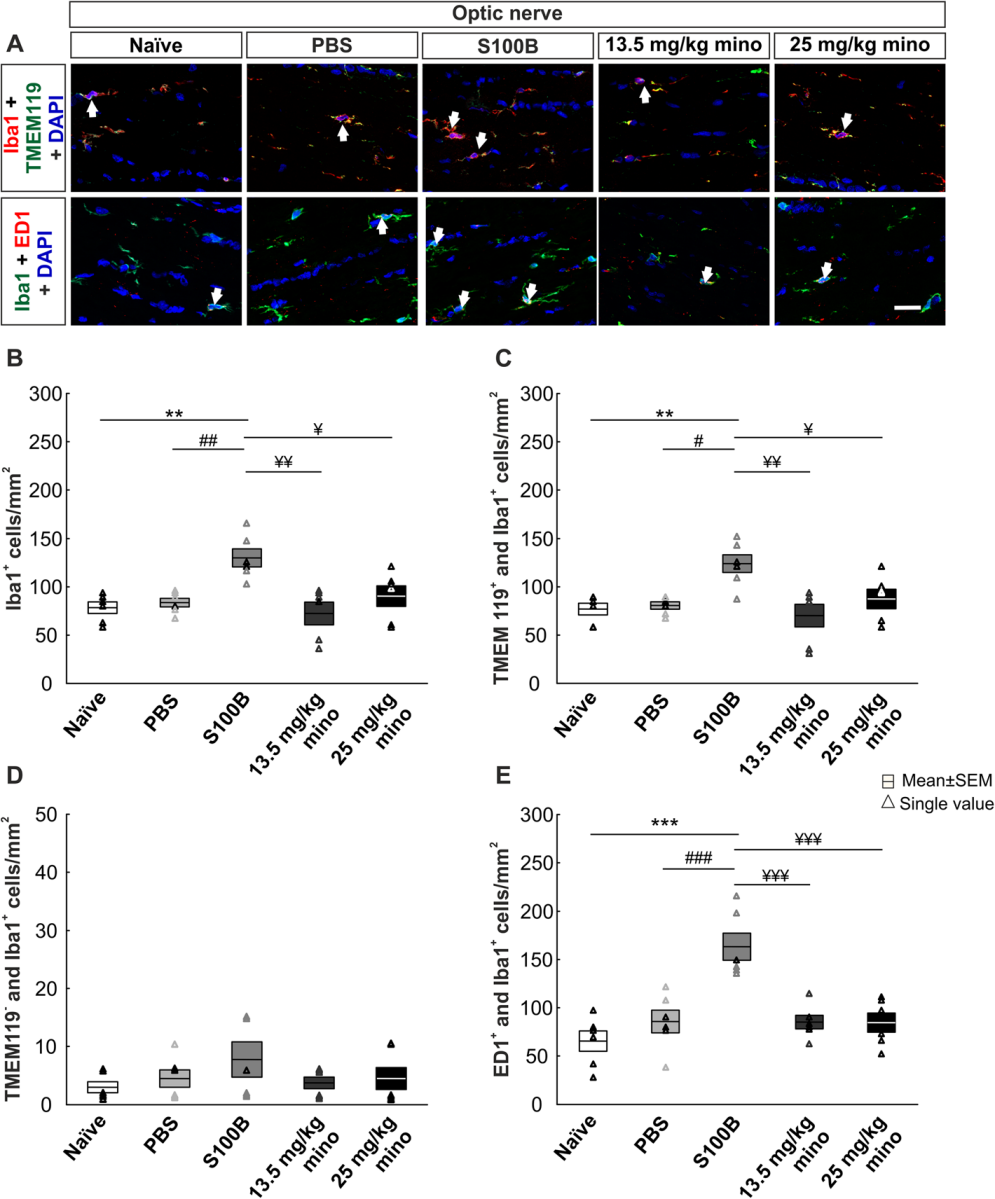
Minocycline application inhibited microglia reaction in optic nerves**. a** Staining of phagocytes (Iba1^+^, red), microglia (Tmem119^+^ and Iba1^+^, green), macrophages (Tmem119^−^ and Iba1^+^), microglia/macrophages in an active state (ED1, red, arrows), and cell nuclei (DAPI, blue) was carried out. **b** The Iba1^+^ phagocyte number was increased in the S100B group compared to the naïve and the PBS group. Minocycline treatment reduced phagocyte proliferation. **c** In the S100B group, the number of microglia was increased compared to the naïve, the PBS, the 13.5 mg/kg mino, and the 25 mg/kg mino group. **d** Comparable phagocyte (Tmem119^−^ and Iba1^+^) counts were seen in all groups. **e** An upregulation of active microglia/macrophages was observed in S100B optic nerves compared to all other groups. Scale bar: 20 μm, *n* = 6/group, **p < 0.01, ***p < 0.001 *vs.* naïve group, ^#^*p* < 0.05, ^##^*p* < 0.01, ^###^*p* < 0.001 *vs.* PBS group, ^¥^*p* < 0.05, ^¥¥^*p* < 0.01; ^¥¥¥^*p* < 0.001 *vs.* S100B group

Regarding the optic nerve phagocytes (Iba1^+^), effects were comparable to the retina (Fig. 5a). The number of Iba1^+^ cells was increased in the S100B group (Fig. 5b; Table 3). The naïve (*p* = 0.003), the PBS (*p* = 0.009), the 13.5 mg/kg mino (*p* = 0.001), and the 25 mg/kg mino group (*p* = 0.03) demonstrated significant lower phagocytes numbers compared to the S100B group.

Also, the number of microglia (Tmem119^+^ and Iba1^+^) was increased in the S100B group. The naïve (*p* = 0.006), the PBS (*p* = 0.01), the 13.5 mg/kg mino (*p* = 0.002), and the 25 mg/kg mino group (*p* = 0.04) displayed significantly lower numbers of microglia than S100B nerves (Fig. 5c; Table 3).

Regarding the number of macrophages (Tmem119^−^ and Iba1^+^), no differences were detected between all groups (Fig. 5d; Table 3).

The number of active phagocytes (ED1^+^ and Iba1^+^) was increased in the S100B group compared to the naïve (*p* < 0.001), the PBS (*p* < 0.001), the 13.5 mg/kg mino (*p* < 0.001), and 25 mg/kg mino group (*p* < 0.001). Active phagocyte counts in all other groups were comparable (Fig. 5e; Table 3).

Therefore, also in the optic nerve, minocycline was able to suppress the S100-induced activation of microglia.

### Unchanged retinal macroglia area

For immunofluorescence analysis of the astrocytes in the retina, we used antibodies against GFAP and S100B (supplemental fig. 2A).

The GFAP signals in the investigated groups did not show any differences (Table 3). Also, the analysis of the S100B area in the retina demonstrated no alterations between the groups.

Astrocytes in the retina do not appear to be affected by S100B.

### Reduced infiltration of inflammatory cells and slight effects of astrocytes in the optic nerve

Infiltration of inflammatory cells is a common occurrence in neuronal degeneration. To observe possible cell infiltration, optic nerve sections were stained with H&E (supplemental fig. 3A).

After 14 days, more cells infiltrated in the S100B-treated optic nerves than in the naïve (*p* = 0.003), in the PBS (*p* = 0.04), and in the 13.5 mg/kg mino nerves (*p* = 0.01, Table 3). The H&E score of the control groups and both minocycline groups was quite similar (supplemental fig. 3B).

Astrocytes were examined with antibodies against GFAP and S100B in the optic nerve (supplemental fig. 3A). However, the analysis of the GFAP signal area showed a significant difference between the naïve and S100B group (*p* = 0.04). No differences were noted between the other groups (supplemental fig. 3C).

In contrast, the S100B signal area was comparable in all groups. In summary, minocycline seems to prevent the infiltration of cells into the optic nerve. The optic nerve astrocytes appear to be only slightly affected by S100B.

### Characterization of retinal proteome changes induced by S100B, 13.5 mg/kg and 20 mg/kg minocycline treatment

**Fig. 6:**
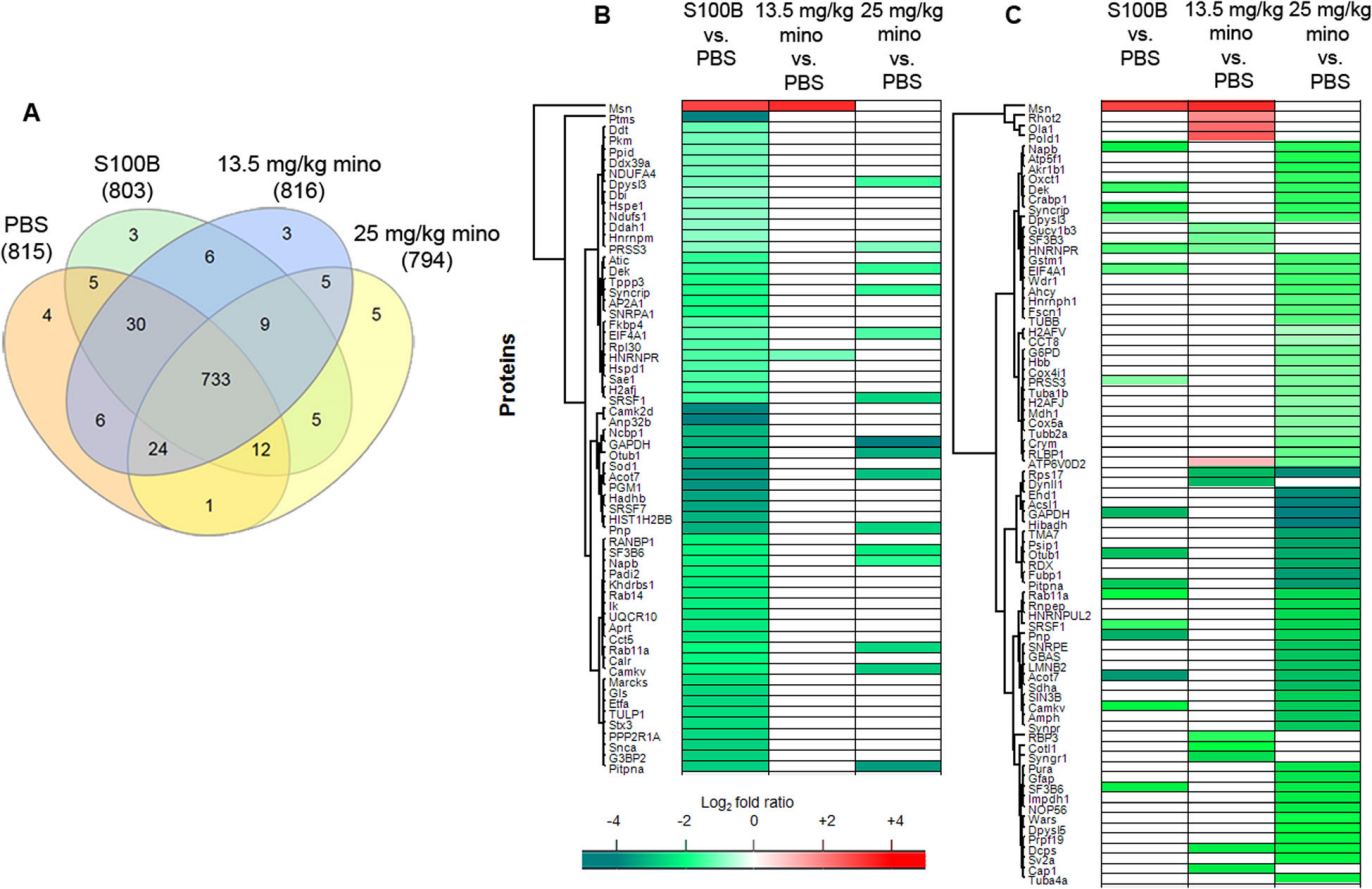
Global retinal proteome underlying the designated treatment paradigms. **a** Venn diagram depicting overlaps of identified retinal proteins in S100B, 13.5 mg/kg mino, 25 mg/kg mino, and PBS group. **b** Heat map depicts the hierarchical clustering of all the differentially abundant retinal proteins based on the log_2_ fold ratio related to S100B. **c** In this heat map, the hierarchical clustering of all the differentially abundant retinal proteins based on the log_2_ fold ratio related to minocycline treatments is shown. The upregulated proteins are shown in red and the downregulated proteins are in green

To further characterize the molecular changes induced by S100B and the potential protective effects of 13.5 mg/kg mino or 25 mg/kg mino treatment, we assessed proteome changes in the retina by employing bottom-up mass spectrometry-based discovery proteomics strategy. Label-free quantification analysis of biological triplicates of the designated groups identified a total of 854 retinal proteins with a false discovery rate (FDR) of 1% (supplemental table 2B). A total of 733 proteins were identified in all the groups, as illustrated in the Venn diagrams in Fig. 6a. Among the identified proteins, 142 were found to be significantly differently abundant between the following comparison analyses: S100B *vs*. PBS (62), 13.5 mg/kg mino *vs*. PBS (15), 25 mg/kg mino *vs*. PBS (65), 13.5 mg/kg mino *vs*. S100B (16), 25 mg/kg mino *vs*. S100B (17), and 25 mg/kg mino *vs*. 13.5 mg/kg mino (24) (supplemental table 3).

Interestingly, large numbers of differentially abundant proteins were found in the S100B *vs*. PBS (decreased abundance: 61 proteins; increased abundance: 1 protein) and 25 mg/kg mino *vs*. PBS groups (decreased abundance: 65 proteins) compared to the 13.5 mg/kg mino *vs*. PBS group (decreased abundance: 10 proteins; increased abundance: 5 proteins). The 62 differentially abundant proteins related to S100B insults compared to the 13.5 mg/kg mino and 25 mg/kg mino treatments (Fig. 6b). Noteworthy, a large number of decreased proteins observed in the S100B group was not observed in the 13.5 mg/kg mino and 25 mg/kg mino groups. However, from the 77 differentially abundant proteins related to the 13.5 mg/kg mino and 25 mg/kg mino treatments compared to the S100B insults are largely exclusively in the 25 mg/kg mino treatment group (Fig. 6c).

### 13.5 mg/kg minocycline treatment decreased the apoptotic, inflammatory, and metabolic processes induced by S100B

The canonical pathway analysis of the differentially abundant proteins in S100B *vs*. PBS demonstrated the association of adenine and adenosine salvage I, sirtuin signaling pathway, mitochondrial dysfunction, glycolysis I, oxidative phosphorylation, and glycogen degradation III (supplemental table 4). On the contrary, differentially abundant proteins in 13.5 mg/kg mino *vs*. PBS were not significantly associated with the aforementioned pathways, except for phagosome maturation. However, differentially abundant proteins in 25 mg/kg mino *vs.* PBS were found to be significantly associated with phagosome maturation, oxidative phosphorylation, 14-3-3-mediated signaling, mitochondrial dysfunction, TCA cycle II, gluconeogenesis I, adenine and adenosine salvage I, sirtuin signaling pathway, and glycolysis I. The differentially abundant proteins in 13.5 mg/kg mino *vs.* S100B were found to be significantly associated with adenine and adenosine salvage I and glycolysis I. Meanwhile, the differentially abundant proteins in 25 mg/kg mino *vs.* S100B were found to be significantly associated with TCA cycle II and glycogen degradation III. Finally, the differentially abundant proteins in 25 mg/kg mino *vs.* 13.5 mg/kg mino were found to be significantly associated with phagosome maturation, TCA cycle II, 14-3-3-mediated signaling, adenine and adenosine salvage I, and sirtuin signaling pathway.

**Fig. 7:**
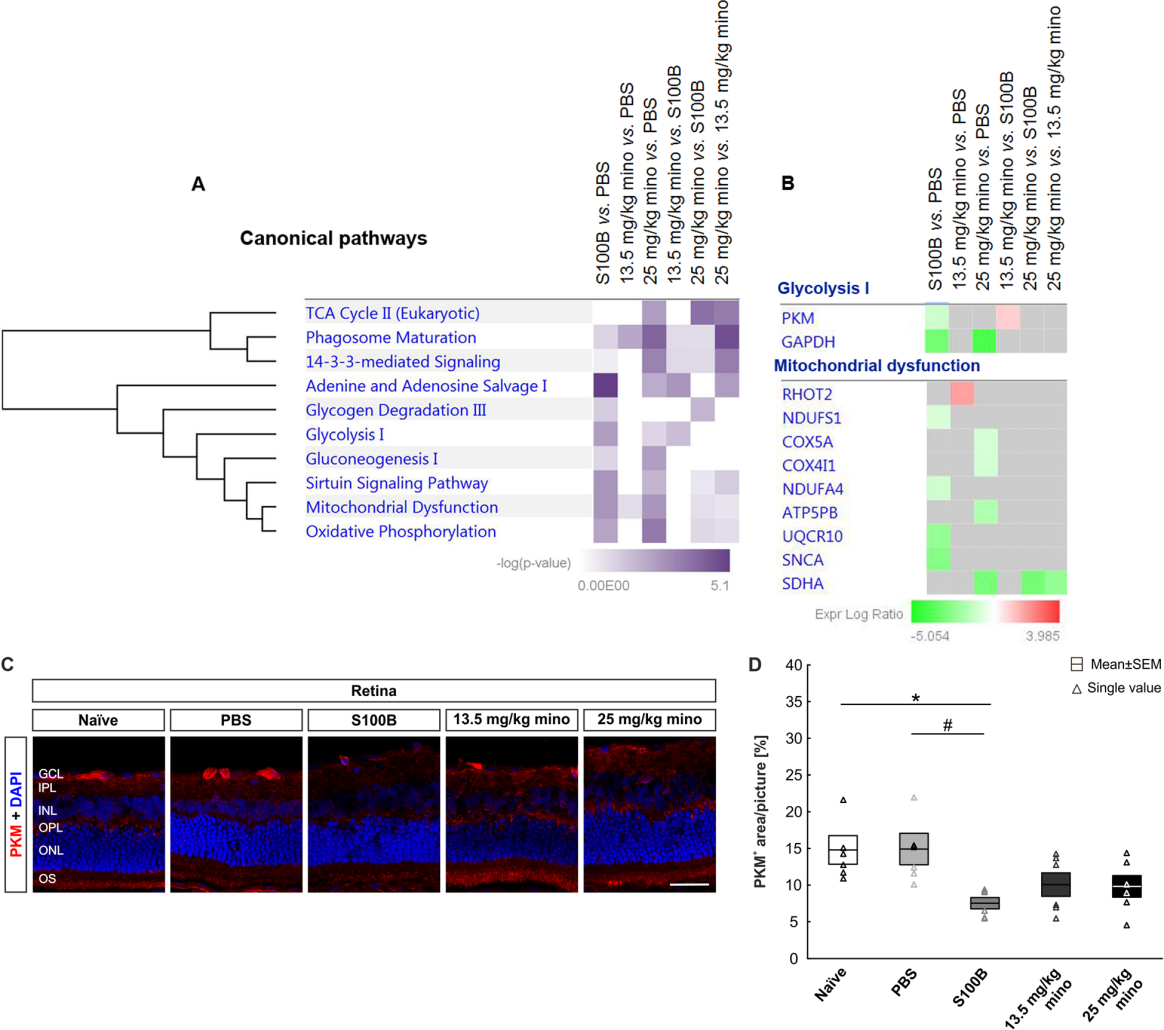
Top canonical pathways annotation analysis of the differentially abundant proteins. **a** Comparison analysis between the designated groups shows the canonical pathways, which were significantly modulated in the retinal samples. Bar charts depict the significantly negative log_10_-transformed *p* values (*p* < 0.05) enriched canonical pathways. **b** Heat maps of proteins associated with glycolysis I and mitochondrial dysfunction in the comparison analysis between the designated groups. Colours red and green represent increment and decrement of protein abundance, respectively, with different colour intensities that correspond to the degree of differential expression. **c** Pyruvate kinase (PKM, red) and cell nuclei (DAPI, blue) were stained in retinal cross-sections. PKM was mainly localized in GCL, INL, INL, and OS. **d** PKM staining area was significantly smaller in the S100B group than in the naïve and PBS group. GCL: ganglion cell layer, IPL: inner plexiform layer, INL: inner nuclear layer, OPL: outer plexiform layer, ONL: outer nuclear layer, OS: photoreceptor outer segments. Scale bar = 20 μm, *n* = 6/group, *p < 0.05 *vs.* naïve group, ^#^*p* < 0.05 *vs.* PBS group

The top disease and biological function analysis of the differentially abundant proteins demonstrated the activation of necrosis, apoptosis, reorganization of cytoskeleton, inflammation of organ, and neurodegeneration in S100B *vs*. PBS (Fig. 7b). Importantly, apoptosis was highly activated in 25 mg/kg mino *vs*. S100B.

**Fig. 8:**
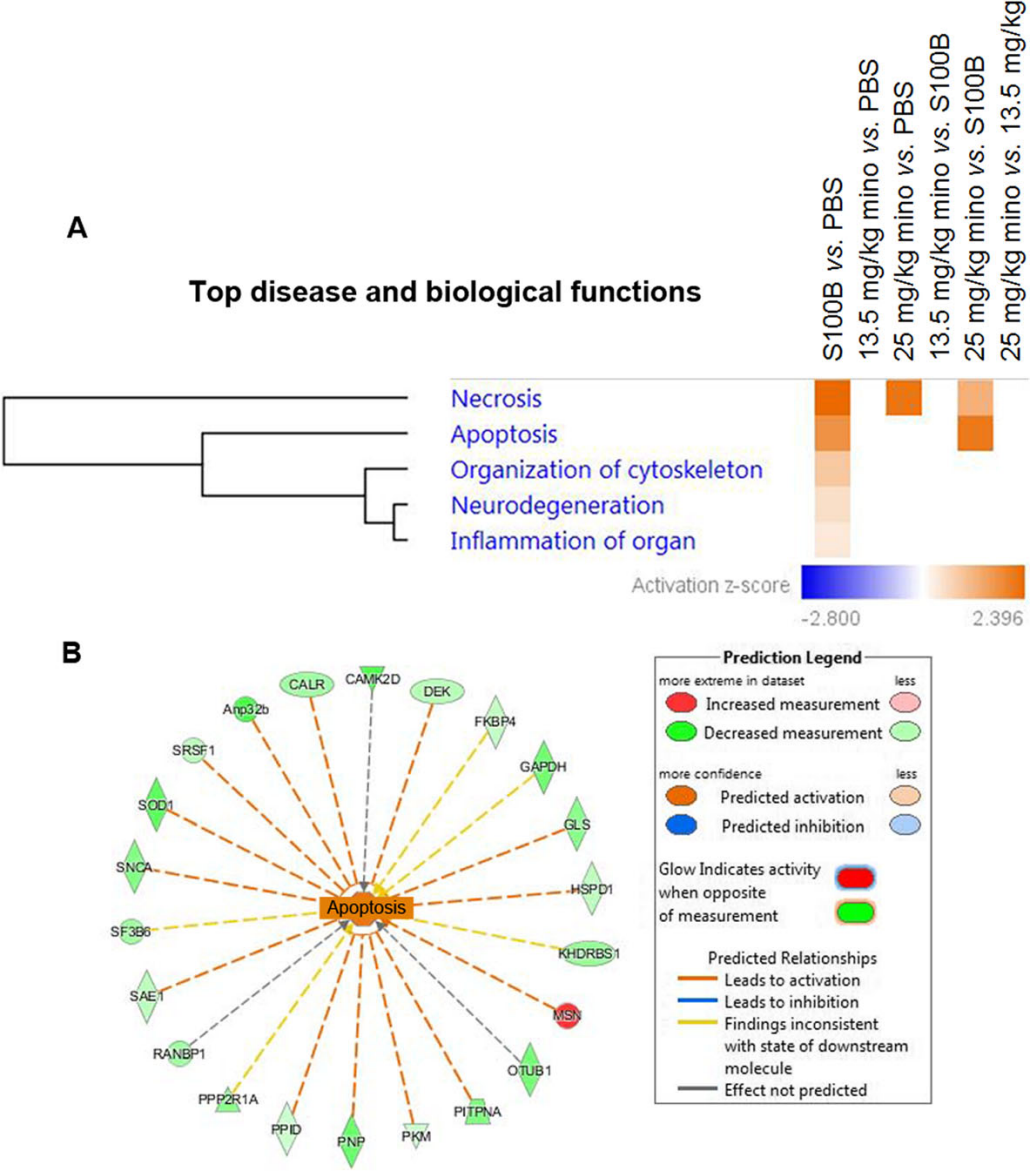
Top diseases and biological function analysis of the differentially abundant proteins. **a** The significantly affected disease and biological functions in the retinal samples associated with the different treatment paradigms. *Z*-scores are represented by the colour orange, which indicates activation, and blue, which indicates inhibition of the associated functions. **b** Network of proteins associated with apoptosis in the comparison analysis between S100B and PBS. Colours red and green represent increment and decrement of protein abundance, respectively, with different colour intensities that correspond to the degree of differential expression. Proteins are depicted as different shapes, which represent the functional classes of the proteins (e.g., enzyme, transporter, ion channel)

Figure 8b displays the 23 proteins involved in the apoptosis process in the S100B *vs*. PBS group, namely, superoxide dismutase (SOD1), Ran-specific GTPase-activating protein (RANBP1), and pyruvate kinase (PKM). In contrast, differentially abundant proteins in 13.5 mg/kg mino *vs.* PBS were not significantly associated with the aforementioned top diseases and biological functions.

**Fig. 9:**
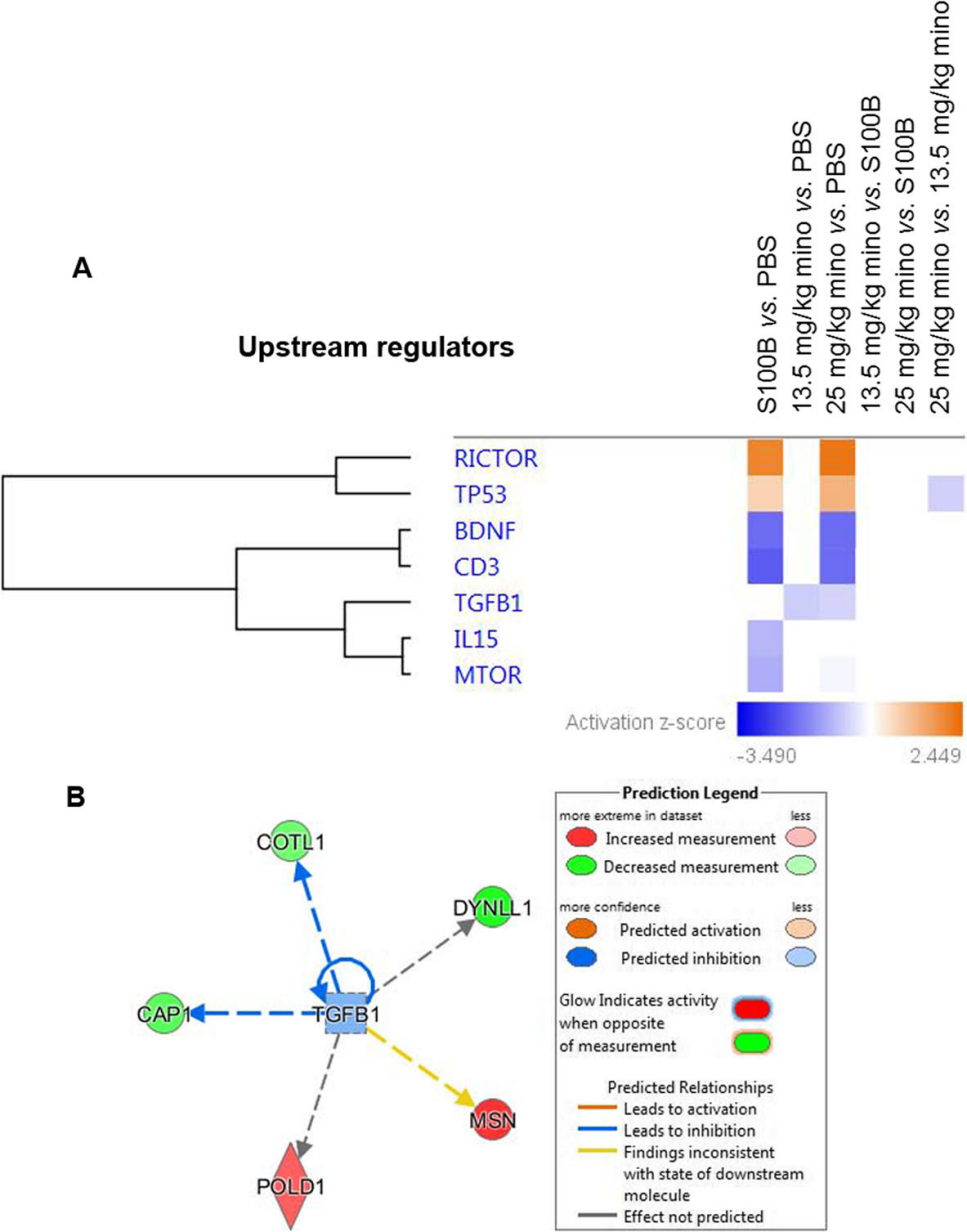
Top upstream regulators analysis of the differentially abundant proteins. **a** The top upstream regulators of the differentially expressed retinal proteins associated with different treatment paradigms. *Z*-scores are represented by the colour orange, which indicates activation, and blue, which indicates inhibition of the associated diseases and biological functions. **b** Network of proteins associated with TGFB1 in the comparison analysis between 13.5 mg/kg mino and PBS. Colours red and green represent increment and decrement of protein abundance, respectively, with different colour intensities that correspond to the degree of differential expression. Proteins are depicted as different shapes, which represent the functional classes of the proteins (e.g., enzyme, transporter, ion channel)

The upstream regulators of the differentially abundant proteins demonstrated the activation of rapamycin-insensitive companion of mammalian target of rapamycin (RICTOR) and tumor suppressor-Proteins p53 (TP53) in S100B *vs*. PBS and 13.5 mg/kg mino *vs*. PBS (Fig. 9; supplemental table 5). On the contrary, the brain-derived neurotrophic factor (BDNF) and cluster of differentiation 3 (CD3) were predicted to be inhibited in S100B *vs*. PBS and 13.5 mg/kg mino *vs*. PBS. Meanwhile, the interleukin 15 (IL15) and mechanistic target of rapamycin (mTOR) were inhibited in the S100B *vs*. PBS group. Interestingly, none of aforementioned upstream regulators were found to be associated with the 13.5 mg/kg mino *vs.* PBS. Importantly, transforming growth factor beta (TGFβ) was found to be only associated with the treatments of 13.5 mg/kg mino and 20 mg/kg mino compared to PBS (Fig. 9b).

### Protein-protein interaction (PPI) of the differentially abundant proteins

**Fig. 10:**
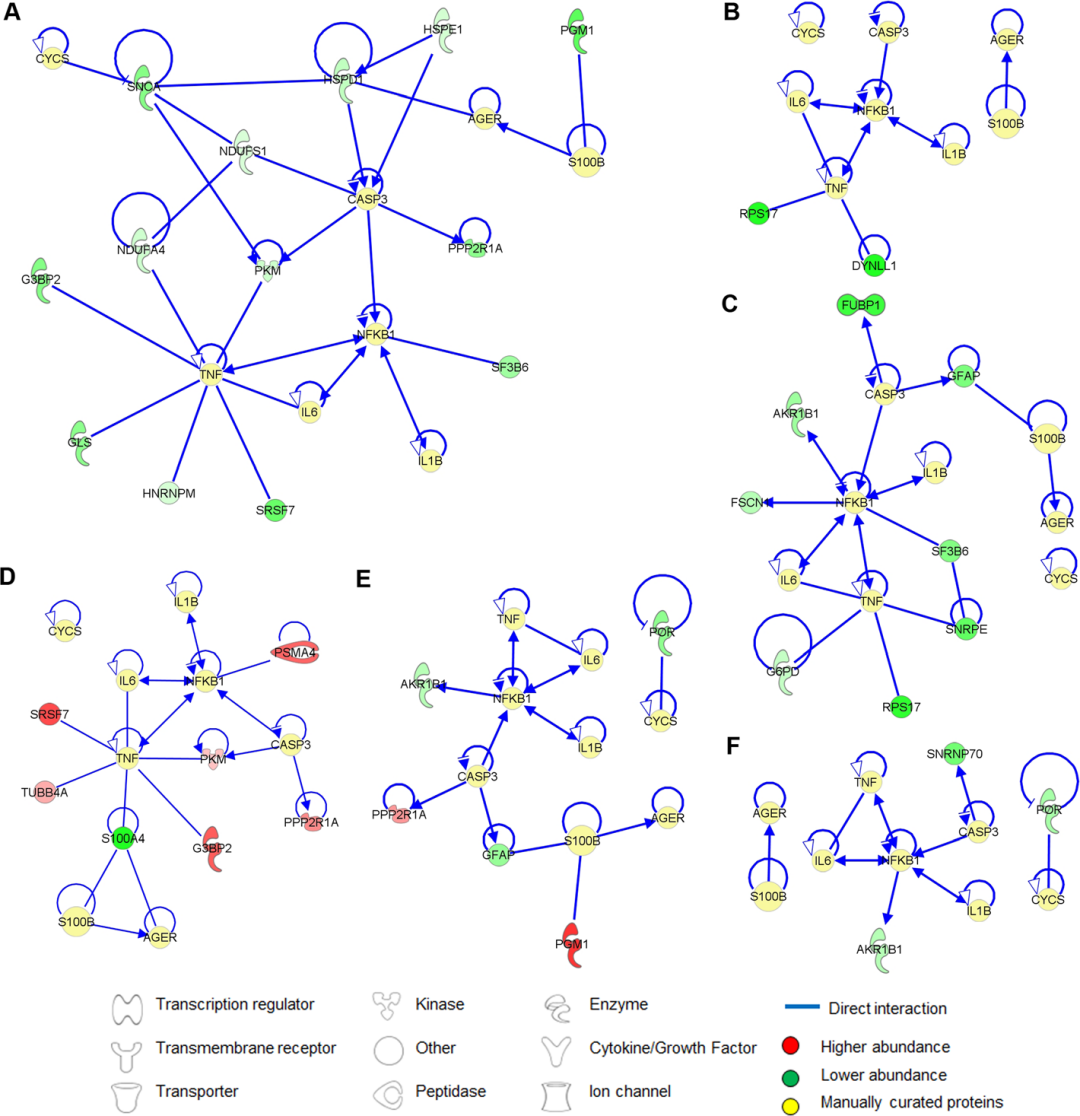
The protein-protein interaction network of differentially expressed retinal proteins in the designated groups with manually curated proteins associated with functionality of the treatment paradigms. **a** S100B *vs.* PBS. **b** 13.5 mg/kg mino *vs.* PBS. **c** 25 mg/kg mino *vs.* PBS. **d** 25 mg/kg mino *vs.* S100B. **e** 25 mg/kg mino *vs.* S100B. **f** 25 mg/kg mino *vs.* 13.5 mg/kg mino. Colours red and green represent increment and decrement of protein abundance, respectively, with different colour intensities that correspond to the degree of differential expression. Proteins are depicted as different shapes, which represent the functional classes of the proteins (e.g., enzyme, transporter, ion channel)

Protein-protein interaction (PPI) networks of all the differentially abundant retinal proteins in the designated groups associated with the manually curated proteins related to the functionality of the treatment’s paradigms (shown in Fig. 10a–f). The manually curated proteins used were established inflammatory and apoptosis markers, comprising cytochrome c (CYCS), advanced glycosylation end product (AGER), S100B, caspase 3 (CASP3), tumor necrosis factor (TNF), NFκB1, IL1β, and IL6. As many as 12, 2, 8, 8, 7, 5, and 3 differentially abundant proteins were associated with the aforementioned in the inflammatory and apoptosis markers in the S100B *vs*. PBS, 13.5 mg/kg mino *vs*. PBS, 25 mg/kg mino *vs*. PBS, 13.5 mg/kg mino *vs*. S100B, 25 mg/kg mino *vs*. S100B, and 25 mg/kg mino *vs*. 13.5 mg/kg mino group, respectively (Fig. 8; supplemental table 4). Notably, for S100B *vs.* PBS, PPIs were observed for S100B with GFAP, CASP3 with GFAP and FUBP1, TNF with G6PD, SNRPF, and RPS17, and NFκB1 with FSCN1, AKR1B1, and SF3B6. However, the aforementioned PPIs were not observed in the 13.5 mg/kg mino *vs*. PBS, except for TNF with RPS17 and DYNLL1. On the contrary, as many as 8 proteins were found to be associated with AGER, CASP3, TNF, NFKB1, and CYCS markers for the 25 mg/kg mino *vs.* PBS.

### Pyruvate kinase staining in the retina

Pyruvate kinase (PKM) was identified by proteomics analysis as one of the most significantly altered protein (Fig. 7b). A distinct reduction of this protein level was demonstrated especially in the S100B group. To identify the PKM location in the retina, an immunofluorescence staining was performed. PKM signals were primarily detected in the GCL, IPL, INL, and outer segment (Fig. 7c).

By examining the PKM area, we noted a significantly smaller PKM^+^ area in the S100B group than in both control groups (naïve *vs.* S100B: *p* = 0.03; PBS *vs.* S100B: *p* = 0.03; Fig. 7d, Table 3). No significant differences were noted in the PKM^+^ area of the minocycline groups in comparison to the control groups. Therefore, S100B seems to have a strong influence on PKM and thus on the regulation of the energy balance of retinal cells.

## Discussion

Previously, we investigated possible pathological mechanisms triggered by intravitreal injection of S100B [8]. Since it remained unclear whether the observed microglial response and its pro-inflammatory modes of action are responsible for the neuronal damage, or whether they are only a side effect, an inhibition of the microglial cells by minocycline was carried out in this study. In this S100B glaucoma-like model we can demonstrate that microglia contribute significantly to neuronal degeneration but are not the sole cause of degeneration.

### Improved signal transmission through low minocycline treatment

The best electrical signal transduction in the ERG analysis was observed in the group with the low minocycline dose. In a mouse ischemia-reperfusion model, a low dose of minocycline (20 mg/kg) reduced the effects of ischemia in the scotopic ERG a- and b-wave as well [39]. Zhang et al. also noted that minocycline has positive effects on electrical signal transduction and photoreceptor survival in a light-induced retinal degeneration model [40]. Minocycline therefore has a high potential to protect neuronal cells in case of damage and to maintain the electrical signal transmission. In contrast, the analysis of PhNR showed no differences between the groups. Possibly no differences could be observed due to the non-optimal measurement conditions, since a scotopic ERG was performed and not a photopic ERG [36, 37].

### The minocycline doses make the difference

Minocycline treatment inhibited microglia cells and reduced the apoptotic mechanisms. In addition, a preservation of neuronal tissue was noted through minocycline therapy but not a complete cell rescue. Both minocycline concentrations saved approximately 20% of the RGCs, which were damaged by S100B. The RGC counts in the minocycline and control groups were not statistically significantly different. Hence, a mild protection by minocycline was detectable.

Furthermore, the low dose of minocycline improved the functionality of the cells in the inner nuclear layer and the photoreceptor layer, which are already damaged 14 days after S100B injection [7]. In addition, the lower minocycline dose protected the optic nerve neurofilament. In contrast, the higher dose did not have a positive effect on the neurofilament. Bosco et al. mentioned the same protective effect for the optic nerves in DBA/2 J mice [16]. The reason for the protection was probably not just microglia inhibition. We observed that minocycline prevented the upregulation of apoptotic proteins, which are strongly effected by S100B. One of them is cleaved caspase 3, which was reduced through minocycline, especially by the lower minocycline dose. This was not that surprising, since it is known that minocycline downregulates pro-apoptotic genes [18] and proteins [41] in glaucoma models.

Therefore, the protective effect of minocycline is dose-dependent in this project, which has also been demonstrated in other studies. Huang et al. examined the effect of 10, 20, 30, 80, and 100 mg/kg minocycline in a mouse ischemia-reperfusion model. In this study, an increased number of RGCs could be observed at 20 and 30 mg/kg minocycline, while the higher concentrations (80 and 100 mg/kg) led to RGC loss [39]. In the same publication, the hypothesis was put forward that high doses of minocycline have a toxic effect on neuronal and non-neuronal retinal cells. Other studies in rabbit eyes showed that intravitreally applied minocycline always exerted toxic effects on retinal cells [42]. The canonical pathway analysis also demonstrated that the high minocycline treatment led to the activation of destructive signaling pathways. Observed changes in the 25 mg/kg mino *vs.* PBS group include phagosome maturation, oxidative phosphorylation, 14-3-3-mediated signaling, mitochondrial dysfunction, TCA cycle II, gluconeogenesis I, adenine and adenosine salvage I, and sirtuin signaling pathways. This means that the number of changed signal pathways of the PBS *vs* 25 mg/kg mino analysis is not different from the S100B *vs* PBS analysis (the meaning of these altered signaling pathways will follow in the chapter: regulatory effect of S100B and minocycline treatment in different cellular pathways).

High concentrations of minocycline therefore do not seem to inhibit the degenerative effects of S100B injection. In contrast, studies with the DBA/2 J mouse showed that treatment with 120 mg/kg minocycline led to an improved survival rate of RGCs [16]. This discrepancy seems to indicate that the dosage of minocycline must be adjusted for each type of application, degeneration model, and organism. This was the reason for the usage of a low (13.5 mg/kg body weight) and high minocycline dose (25 mg/kg body weight) for our S100B glaucoma-like model.

### Minocycline inhibited microglia and inflammation

Currently, the microglial response is considered to be a hallmark of neuroinflammation in the central nervous system. In addition, microglia seem to initiate the immune response during glaucomatous neurodegeneration [43]. In the DBA/2J mouse model, a microglia reaction was observed prior to neuronal damage [44]. For this reason, it is necessary to clarify whether the increased microglia response is a direct cause of neuronal degeneration or can be regarded as a side effect. In our intravitreal S100B model and in other glaucoma-like models, the microglia are activated. Due to Tmem119 and Iba1 double staining, we can conclude that the majority of phagocytes in our model are microglia and non-infiltrated macrophages.

We recently observed that S100B increased the amount of NFκB and IL1β. This indicates that microglia were active in a pro-inflammatory manner [8]. The minocycline treatment suppressed the infiltration of immune cells in the retina and optic nerve. The low minocycline dose was more effective in reducing microglia activity in the retina and in the optic nerve. In vitro studies on murine BV-2 microglia cells and primary microglia cells suggest that minocycline prevents lipopolysaccharide (LPS)-stimulated degradation of IkappaBalpha (IκBα), which may lead to inhibition of NFκB transcriptional activity [45]. This is a possible reason for the suppression of microglia activity in our model, since the S100B/RAGE pathway acts via NFκB activation [9]. In case minocycline inhibits the translocation of NFκB, no IL-1β will be produced and microglia are not activated in a pro-inflammatory manner, which also excludes a proliferation of these cells. In addition, minocycline has been reported to suppress the p38 MAPK-mediated proliferation of microglia [46]. Minocycline therefore reduces both the activation and the proliferation of microglia.

A reduction of a S100B triggered pro-inflammatory environment through minocycline was confirmed by our proteomics analysis. Other groups detected a weakening of the mRNA expression of inflammatory genes after minocycline treatment [45, 47]. Interestingly, minocycline reduced the activity of microglia, but it increased the phagosome maturation process of the microglia in our model (Fig. 7). The phagocytosis is an important and complex function of microglia to remove dead and dying cells in the brain mostly following an inflammatory processes [48]. The remaining microglia in the minocycline animals seem to be able to digest apoptotic cells better than in the S100B group. One explanation for this could be that a high proportion of energy is required for phagocytosis [49]. Since glycolysis is altered through S100B, the phagocytosis could also be disturbed.

### Regulatory effect of S100B and minocycline treatment in different cellular pathways

Further regulatory effects were identified by our proteome investigations. Thus far, regarding the general retina proteome, hundreds of proteins have been identified for rat [50–52], mouse [22, 53–55], monkey [56–59], and human [21, 60, 61]. Yet, studies directing on the retina proteome in glaucoma are still limited [21]. Among the 854 proteins identified in the rat retina, we identified as many as 142 proteins to be differentially abundant with S100B-induced insult and minocycline treatments compared to PBS. To our knowledge, this is the first study of a retinal proteome in an experimental model of S100B-induced retinal insult and evaluation of the efficacy of minocycline treatments thereafter. Unraveling of the significant alterations in numerous metabolic functions, cellular stress, and corresponding diseases, including adenine and adenosine salvage I, sirtuin signaling pathway, mitochondrial dysfunction, glycolysis I, oxidative phosphorylation, glycogen degradation III, apoptosis, organization of cytoskeleton, inflammation, and neurodegeneration, supports the association of a range of molecular changes involved in S100B-induced damage in the retina.

These altered signaling pathways can be another reason for the increased degeneration after the S100B injection. For example, the sirtuin pathway was upregulated through S100B. Sirtuins are implicated in influencing cellular processes, like aging, apoptosis, inflammation, mitochondrial activity, and glucose metabolisms [62]. Interestingly, NFκB is also a target of sirtuins [63]. Sirtuins are important for the mitochondrial activity as well, which is disturbed in the S100B retina. It is known that proteins of the sirtuin signaling pathway, such as SIRT3, are located in the mitochondrial matrix and act as metabolic sensors [64]. SIRT3 reacts to changes in the energy status of cells and regulates the activity of the important metabolic enzymes by modelling protein deacetylation [65]. Studies indicate that SIRT3 also plays a pro-apoptotic role in the BCL2-53 and Jun N-terminal kinase-regulated apoptosis pathway [66]. The upregulation of the sirtuin signaling pathway by S100B could therefore increase degeneration in retina and optic nerve.

Another degenerative factor detected by proteome analysis is mitochondrial dysfunction. A mitochondrial dysfunction leads to an energy and oxidative stress problem [67]. It is known that persistent activation of RAGE by S100B produces increased amounts of oxygen radicals, which could then lead to mitochondrial dysfunction [68, 69]. Consequently, metabolic processes like glycolysis, oxidative phosphorylation, and glycogen degradation III were altered in the S100B group. For example, it appears that S100B injection leads to dysfunction in certain glycolysis proteins. A strongly decreased protein concentration of pyruvate kinase (PKM) was found in the S100B group. PKMs are glycolytic enzymes that transfer a phosphate group from phosphoenolpyruvate to adenosine diphosphate generating pyruvate and adenosine triphosphate. For this reason, they are essential for energy generation [70]. In the retina, PKM is expressed in the cell bodies and synaptic terminals of photoreceptors as well as in the inner retinal neurons [71]. Our histological examination revealed PKM^+^ signals in GCL, IPL, INL, and photoreceptor outer segments (OS) in all five groups. In accordance with the proteomic analysis, the histological evaluation revealed a significantly smaller PKM^+^ staining area in the S100B group. Therefore, glycolysis seems to be disrupted through S100B. Another essential protein for glycolysis is the protein glyceraldehyde-3-phosphate dehydrogenase (GAPDH). This enzyme catalyzes the dehydration of glyceraldehyde-3-phosphate into 1,3-bisphosphoglycerate during glycolysis [72]. This protein is strongly downregulated in the S100B group, which additionally indicates a disruption of glycolysis by S100B.

Another degeneration hallmark is the degradation of RNA and DNA. The nucleotide salvage pathways are important to recover nucleosides that are formed during the degradation [73]. This is the reason for the strong increase of the adenine and adenosine salvage I pathway in the S100B retinas. The alterations of glycolysis and adenine and adenosine salvage I pathway were reduced through the lower minocycline dose, while the high minocycline dose reduced only the glycogen degradation. The lower dosage of minocycline showed stronger protective effects here, too. Generally, the exploratory proteomics approach in this study has unraveled the complex mechanism of S100B-induced insult in the retina, which provides vast opportunities for better in-depth understanding of the potential protective effects of compounds, such as minocycline, as demonstrated in this study.

## Conclusion

**Fig. 11:**
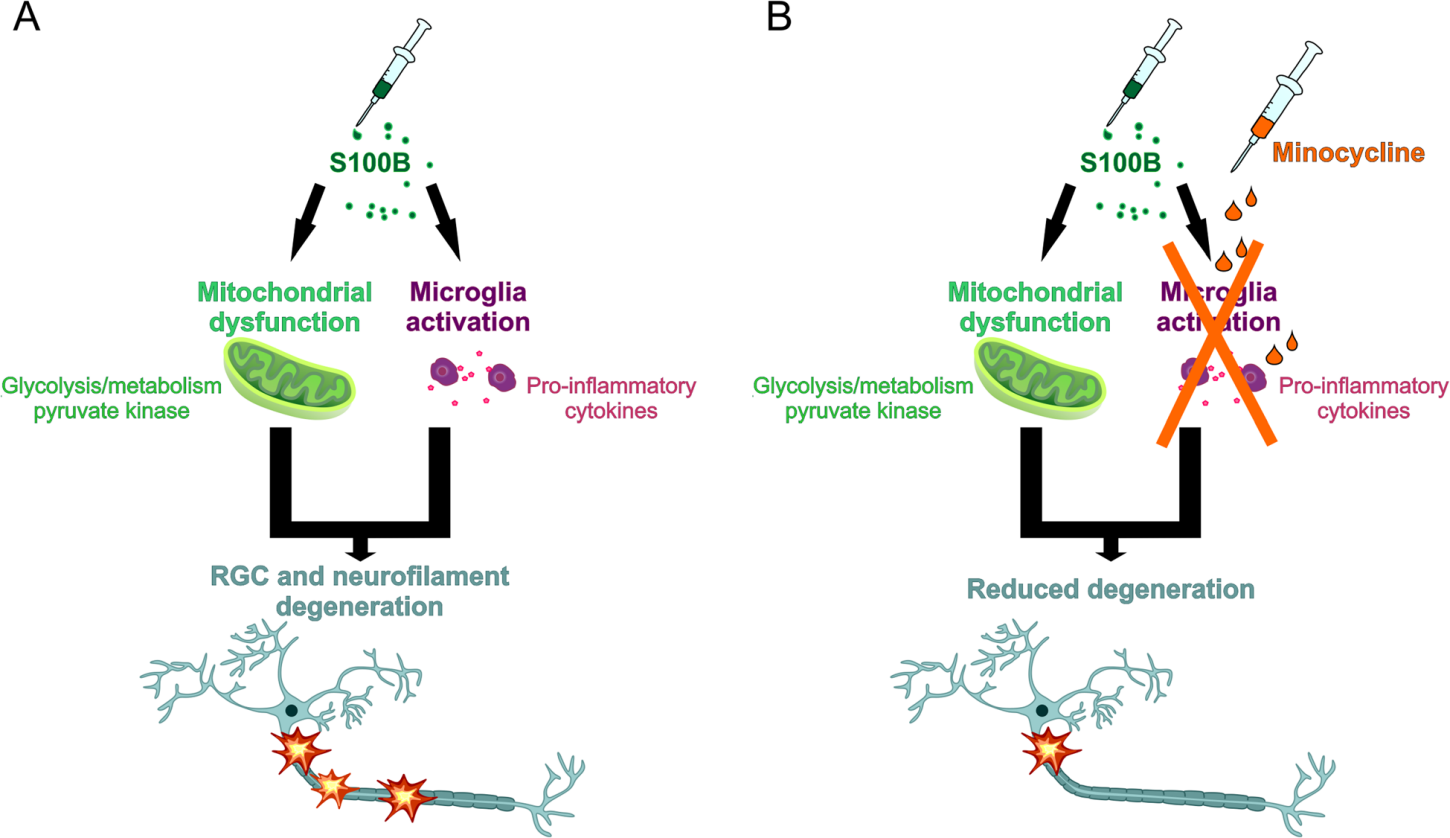
Schematic representation of the effects of S100B and minocycline in this model. **a** Intravitrially injected S100B induced a pro-inflammatory response that included increased activation of microglia and release of inflammatory cytokines. Mass spectrometric proteome analysis indicates that S100B also caused mitochondrial dysfunction, which disrupts cellular metabolism and energy production. These two factors led to RGC degeneration and neurofilament loss. **b** Treatment with minocycline blocked microglia activation. Therefore, some RGCs were protected and the neurofilament remained largely intact. The apoptosis rate was also lowered. Neuronal damage was minimized, but not completely inhibited, since mitochondrial dysfunction and altered glycolysis have been induced despite minocycline treatment

Both minocycline doses were able to reduce the microglial activity, which led to a less inflammatory environment (Fig. 11). However, a stronger protective and regulative effect was observed with the lower dose. We assume that the degeneration through S100B had several causes and the microglia response was one of the main, but not the only one. The activation of apoptosis, the sirtuin pathway, mitochondrial dysfunction, and the energy and oxidative stress problems are involved in the S100B-mediated degeneration process. Minocycline, especially in the low concentration, could reduce some of these processes, which protected RGCs and optic nerve neurofilament. We conclude that microglia have a strong influence on the degeneration in this model, but they are not solely responsible.

## Supplementary Information

**Additional file 1:** **Supplementary table 1.** Experimental design of proteomics analysis.

**Additional file 2:** **Supplementary table 2a.** MaxQuant parameters. **Supplementary table 2b.** List of proteins identified employing MaxQuant (*Rattus and Homo sapiens*).

**Additional file 3:** **Supplementary table 3.** Differentially expressed proteins in the designated groups.

**Additional file 4:** **Supplementary table 4A.** Protein-protein relations types of the differentially expressed proteins in S100B *vs.* PBS. **Supplementary table 4B.** Protein-protein relations types of the differentially expressed proteins in 13.5 mg/kg mino *vs.* PBS. **Supplementary table 4C.** Protein-protein relations types of the differentially expressed proteins in 25 mg/kg mino *vs.* PBS. **Supplementary table 4D.** Protein-protein relations types of the differentially expressed proteins in 13.5 mg/kg mino *vs.* S100B. **Supplementary table 4E.** Protein-protein relations types of the differentially expressed proteins in 25 mg/kg mino *vs.* S100B.** Supplementary table 4F.** Protein-protein relations types of the differentially expressed proteins in 25 mg/kg mino vs. 13.5 mg/kg mino.

**Additional file 5:** **Supplementary table 5.** Functional annotation analysis of the differentially abundant proteins.

**Additional file 6:** **Supplemental figure 1.** Slight improvement in electrical signal transmission after minocycline treatment. A) Retinal functionality was investigated through ERG measurements. In regard to the a-wave amplitudes, the highest values were noted in the 13.5 mg/kg mino group. Significant differences between the five groups were only observed at 0.1, 10, and 25 cd.s/m^2^. At 0.1 cd.s/m^2^, the 13.5 mg/kg mino group had an higher a-wave amplitude than the S100B group. Also, the 25 mg/kg mino group demonstrated higher amplitudes then the S100B group. At 10 cd.s/m^2^, 13.5 mg/kg mino group showed higher values then the S100B group. The same was observed at 25 cd.s/m^2^. The S100B animals displayed lower amplitudes then the 13.5 mg/kg mino and naïve group. B) The analysis of the b-wave showed significant differences at 0.3 and 1 cd.s/m^2^. At 0.3 and 1 cd.s/m^2^, the 13.5 mg/kg mino group displayed significantly higher amplitudes than the S100B group. 1 cd.s/m^2^, the naïve group had higher values than the S100B group. C) No differences between the five groups were observed regarding the photopic negative response (PhNR) at 0.1 cd/m^2^.*p < 0.05 *vs.* naïve group, ^*#*^*p* < 0.05 *vs.* PBS group, ^¥^*p* < 0.05, ^¥¥^*p* < 0.01 *vs.* S100B group.

**Additional file 7:** **Supplemental figure 2.** Comparable macroglia area in all groups. A) Retinal macroglia were evaluated with immunofluorescence to mark GFAP (red) and S100B (green) and cell nuclei were stained with DAPI (blue). B) The GFAP^+^ area was similar in all groups. C) Also, the S100B^+^ area was very similar in all groups. Abbreviations: GCL: ganglion cell layer, IPL: inner plexiform layer, INL: inner nuclear layer, ONL: outer nuclear layer. Scale bar: 20 μm, n = 6/group.

**Additional file 8:** **Supplemental figure 3.** Higher cell infiltration in S100B optic nerves and slihth effected macroglia. A) The structure of the optic nerve was visualized through H&E staining. The macroglia area was visualized with GFAP (red) and S100B (green) and cell nuclei with DAPI (blue). B) S100B significantly increased cell infitration in the optic nerves when compared to controls. The lower dose of minocycline reduced this infiltration significantly. C) The GFAP^+^ area in the S100B group was increased compared to the naïve group. D) The S100B^+^ area was similar in all groups. Scale bar = 20 μm, n = 6/group, *p < 0.05, **p < 0.01 *vs.* naïve group, ^*#*^*p* < 0.05 *vs.* PBS group, ^¥^*p* < 0.05 *vs.* S100B group.

## Data Availability

All the information pertaining to the proteomic study, especially the experimental design, search parameters, raw data, and statistical information of the *p* value and log ratio differences as well as the functional annotation analysis were presented fully and available in the supplementary information.

## Abbreviations

AGER: Advanced glycosylation end product
CASP3: Caspase 3
CYCS: Comprising cytochrome c
GAPDH: Glyceraldehyde-3-phosphate dehydrogenase
GCL: Ganglion cell layer
GOCC: Gene ontology cellular component
IL: Interleukin
INL: Inner nuclear layer
IPL: Inner plexiform layer
LPS: Lipopolysaccharide
NFκB: Nucleus factor-kappa-light-chain-enhancer of activated B cells
PKM: Pyruvate kinase
PPI: Protein-protein interaction
ONL: Outer nuclear layer
OPL: Outer plexiform layer
OS: Photoreceptor outer segments
PBS: Phosphate-buffered saline
RAGE: Receptor for advanced glycation endproducts
RGC: Retinal ganglion cell
TNF: Tumor necrosis factor
