# Supplementary figures and images for: Minocycline reduces inflammatory response and cell death in a S100B retina degeneration model
Source: J Neuroinflammation. 2020 Dec 14;17:375. doi: 10.1186/s12974-020-02012-y (PMC7737388; doi:10.1186/s12974-020-02012-y)

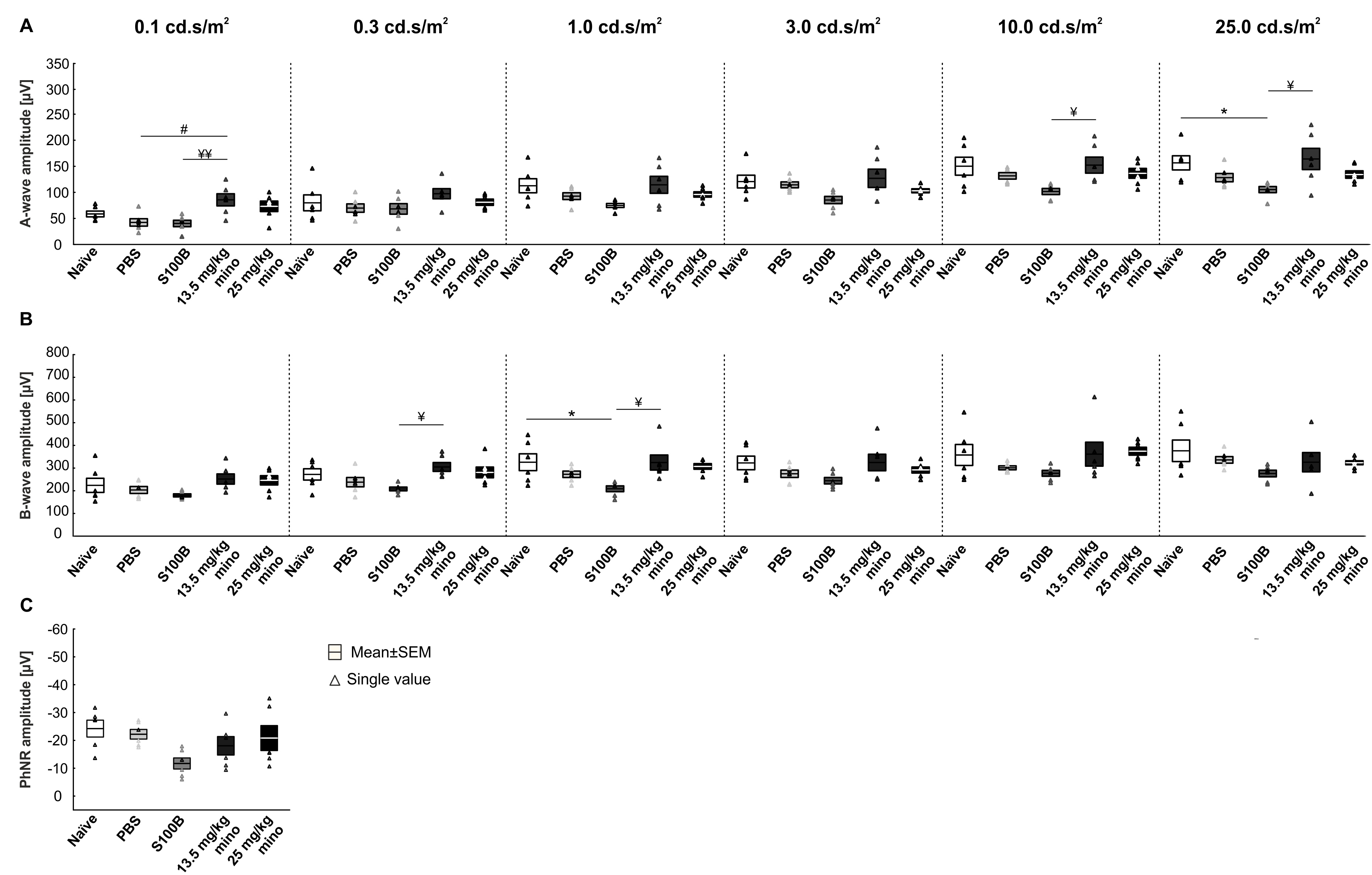

Supplement: Supplementary file 6 — Additional file 6: Supplemental figure 1. Slight improvement in electrical signal transmission after minocycline treatment. A) Retinal functionality was investigated through ERG measurements. In regard to the a-wave amplitudes, the highest values were noted in the 13.5 mg/kg mino group. Significant differences between the five groups were only observed at 0.1, 10, and 25 cd.s/m2. At 0.1 cd.s/m2, the 13.5 mg/kg mino group had an higher a-wave amplitude than the S100B group. Also, the 25 mg/kg mino group demonstrated higher amplitudes then the S100B group. At 10 cd.s/m2, 13.5 mg/kg mino group showed higher values then the S100B group. The same was observed at 25 cd.s/m2. The S100B animals displayed lower amplitudes then the 13.5 mg/kg mino and naïve group. B) The analysis of the b-wave showed significant differences at 0.3 and 1 cd.s/m2. At 0.3 and 1 cd.s/m2, the 13.5 mg/kg mino group displayed significantly higher amplitudes than the S100B group. 1 cd.s/m2, the naïve group had higher values than the S100B group. C) No differences between the five groups were observed regarding the photopic negative response (PhNR) at 0.1 cd/m2.*p < 0.05 vs. naïve group, #p < 0.05 vs. PBS group, ¥p < 0.05, ¥¥p < 0.01 vs. S100B group. [file 12974_2020_2012_MOESM6_ESM.tif]

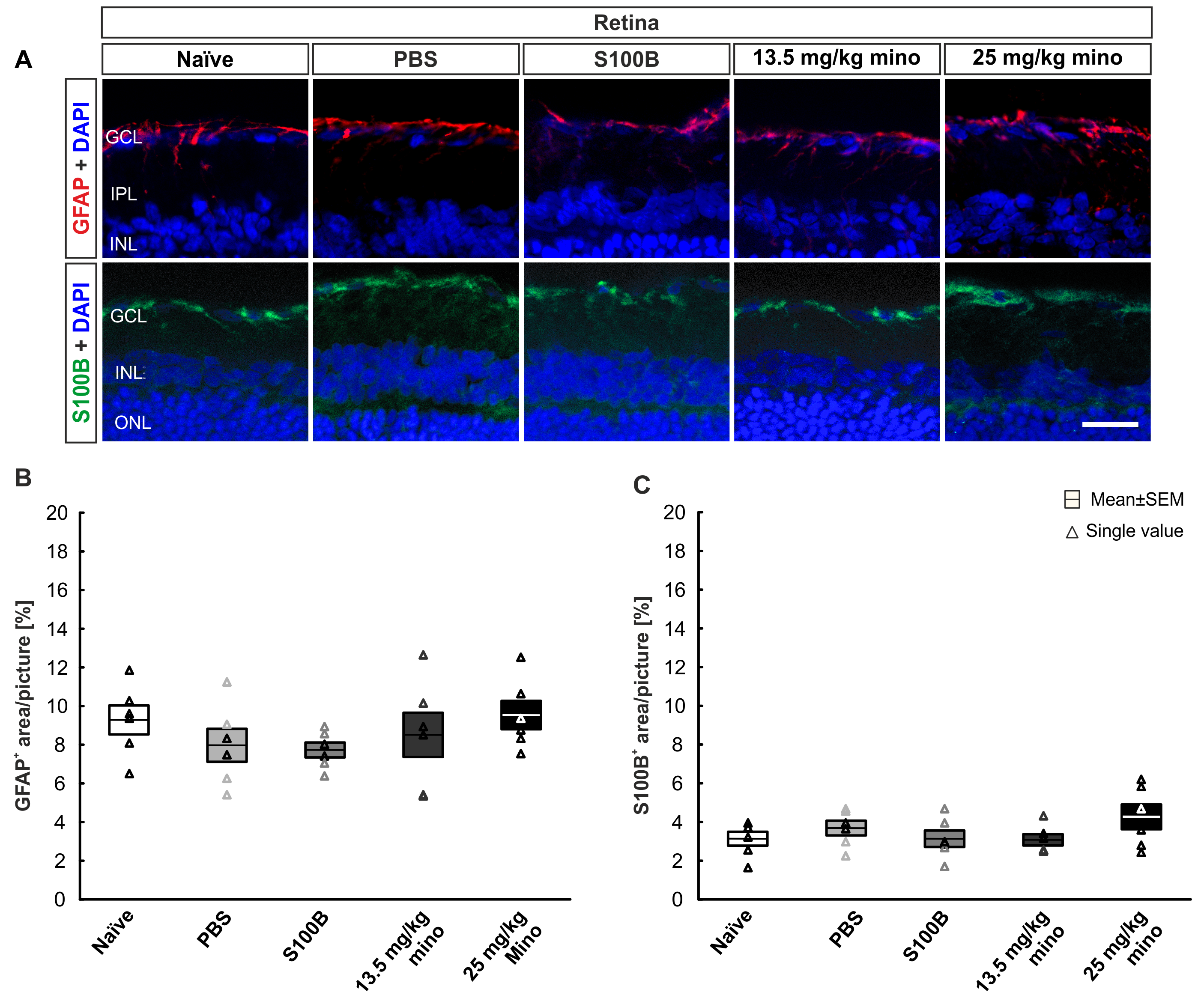

Supplement: Supplementary file 7 — Additional file 7: Supplemental figure 2. Comparable macroglia area in all groups. A) Retinal macroglia were evaluated with immunofluorescence to mark GFAP (red) and S100B (green) and cell nuclei were stained with DAPI (blue). B) The GFAP+ area was similar in all groups. C) Also, the S100B+ area was very similar in all groups. Abbreviations: GCL: ganglion cell layer, IPL: inner plexiform layer, INL: inner nuclear layer, ONL: outer nuclear layer. Scale bar: 20 μm, n = 6/group. [file 12974_2020_2012_MOESM7_ESM.tif]

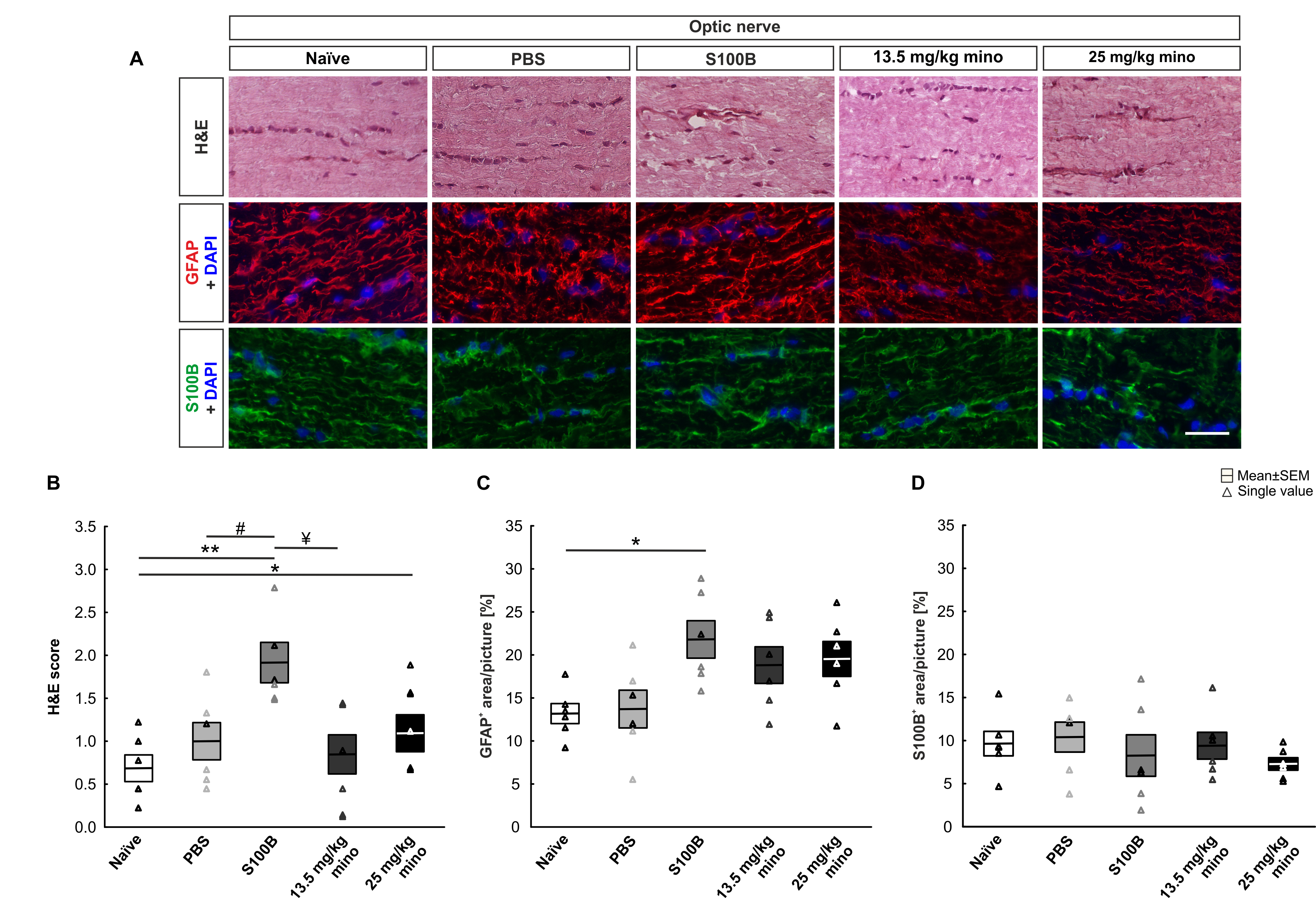

Supplement: Supplementary file 8 — Additional file 8: Supplemental figure 3. Higher cell infiltration in S100B optic nerves and slihth effected macroglia. A) The structure of the optic nerve was visualized through H&E staining. The macroglia area was visualized with GFAP (red) and S100B (green) and cell nuclei with DAPI (blue). B) S100B significantly increased cell infitration in the optic nerves when compared to controls. The lower dose of minocycline reduced this infiltration significantly. C) The GFAP+ area in the S100B group was increased compared to the naïve group. D) The S100B+ area was similar in all groups. Scale bar = 20 μm, n = 6/group, *p < 0.05, **p < 0.01 vs. naïve group, #p < 0.05 vs. PBS group, ¥p < 0.05 vs. S100B group. [file 12974_2020_2012_MOESM8_ESM.tif]
